# Molecular Basis of Behaviorally Active Terpenoid Volatile Recognition by Odorant-Binding Proteins in *Tomicus pilifer*

**DOI:** 10.3390/insects17080810

**Published:** 2026-08-04

**Authors:** Yanan Luo, Sha Hua, Longzheng Wang, Shanchun Yan, Qi Wang

**Affiliations:** 1 Key Laboratory of Sustainable Forest Ecosystem Management, Ministry of Education, Northeast Forestry University, Harbin 150040, China; 18886679539@163.com (Y.L.); wanglzchina@163.com (L.W.); yanshanchun@126.com (S.Y.); 2 Forest Protection Research Institute of Heilongjiang Province, Heilongjiang Academy of Forestry, Harbin 150040, China; huasha0915@126.com

**Keywords:** *Tomicus pilifer*, odorant-binding protein, hindgut and frass volatiles, terpene perception, chemical communication

## Abstract

*Tomicus pilifer* is an important wood-boring forest pest in China, and its host localization and intraspecific communication rely on the perception of volatile chemical cues. However, the molecular mechanisms underlying odor recognition in this species remain largely unknown. In this study, we systematically investigated the behaviorally active volatiles present in the hindgut and feces of *T. pilifer* and elucidated the roles of odorant-binding proteins (OBPs) in their recognition. Gas chromatography–mass spectrometry (GC–MS) identified eight volatile compounds common to both hindgut and fecal samples. Among them, five terpenoid compounds, α-pinene, 3-carene, D-limonene, camphene, and β-myrcene, elicited significant electroantennogram (EAG) responses and induced positive behavioral attraction in adults. Based on antennal transcriptome data, phylogenetic relationships with functionally characterized homologous OBPs, preliminary molecular docking analyses, and tissue-specific expression patterns, three candidate OBPs (TpilOBP5, TpilOBP16, and TpilOBP29) were selected from 51 identified TpilOBP genes and subsequently expressed as recombinant proteins. Fluorescence competitive binding assays demonstrated that all three OBPs bound to the five behaviorally active terpenoid volatiles, with TpilOBP29 exhibiting the broadest ligand-binding spectrum and the highest binding affinity. Molecular docking and interaction analyses further revealed that the binding pocket of TpilOBP29 forms a continuous hydrophobic core composed of multiple conserved hydrophobic residues, which cooperatively stabilizes ligand binding through hydrophobic interactions, π–alkyl interactions, and van der Waals forces, thereby conferring broad-spectrum and high-efficiency odorant recognition. These findings provide new insights into the molecular mechanisms underlying the recognition of key behaviorally active terpenoid volatiles in *T. pilifer*, identify TpilOBP29 as a key mediator of odor recognition, and provide a potential molecular target for the development of environmentally friendly semiochemical-based behavioral management strategies against bark beetle pests.

## Simple Summary

*Tomicus pilifer* is an important forest pest in China. Its host localization and intraspecific communication rely on the perception and recognition of volatile chemical signals; however, the molecular mechanisms underlying olfactory recognition remain poorly understood. In this study, we identified eight volatile compounds shared between the hindgut and frass of *T. pilifer*. Among them, five terpene compounds, including α-pinene, 3-carene, D-limonene, camphene, and β-myrcene, showed significant electrophysiological activity and behavioral attraction to adult beetles. Furthermore, three candidate odorant-binding proteins (TpilOBP5, TpilOBP16, and TpilOBP29) were identified. These proteins were highly expressed in antennae and exhibited stable binding abilities toward the five behaviorally active terpene volatiles. Notably, TpilOBP29 displayed the broadest ligand-binding spectrum and the strongest binding affinity. Structural analysis suggested that its hydrophobic binding pocket, composed of conserved hydrophobic residues, facilitates ligand stabilization through hydrophobic interactions, π–alkyl interactions, and van der Waals forces. Our findings provide new insights into the molecular basis of volatile recognition in *T. pilifer* and identify TpilOBP29 as a potential olfactory target for understanding bark beetle chemical communication and developing semiochemical-based environmentally friendly pest management strategies.

## 1. Introduction

The insect olfactory system plays a pivotal role in the detection of chemical cues and the regulation of ecologically important behaviors, including foraging, mate finding, host recognition, and oviposition site selection [1,2]. Host-derived volatiles and pheromones constitute major semiochemicals that mediate these behaviors by conveying information about food resources, mates, and suitable habitats [3]. Increasing evidence has demonstrated that specific odorants can elicit distinct behavioral responses in insects. For example, larvae of the hoverfly Eupeodes corollae utilize aphid-derived (E)-β-farnesene to locate prey at short range, whereas adults exploit the same compound released by plants to identify aphid-infested hosts over longer distances [4]. Similarly, female red palm weevils, Rhynchophorus ferrugineus, exhibit significant behavioral attraction and antennal responses to trans,trans-2,4-nonadienal and trans-2-nonenal emitted from young coconut leaves [5]. These findings highlight the critical role of odor-mediated communication in insect ecology and underscore the importance of elucidating the molecular mechanisms underlying olfactory perception.

Odor perception in insects is primarily mediated by the antennae, which are densely covered with olfactory sensilla specialized for detecting environmental chemical cues [6,7]. After entering the sensillar lymph, volatile compounds are transported by peripheral olfactory proteins to olfactory receptors, triggering neuronal signal transduction and subsequent behavioral responses [8,9,10]. Among these proteins, odorant-binding proteins (OBPs) are considered key mediators of odor recognition because they bind and transport hydrophobic odorants through the aqueous sensillar lymph to receptor sites [11,12,13]. Increasing evidence has demonstrated that OBPs are involved in the perception of host plant volatiles, pheromones, host localization, and chemical communication [10,13,14]. For example, RpedOBP1 from *Riptortus pedestris* binds the aggregation pheromone (E)-2-hexenyl (Z)-3-hexenoate and contributes to pheromone perception [3], whereas SfruGOBP2 from *Spodoptera frugiperda* exhibits binding affinity toward multiple female-produced sex pheromone components [15]. These findings highlight the pivotal role of OBPs in insect chemoreception and emphasize the importance of elucidating the molecular interactions between OBPs and behaviorally active semiochemicals.

Pheromones serve as key mediators of intraspecific chemical communication in insects and play essential roles in regulating behaviors such as mate recognition, aggregation, dispersal, and oviposition site selection [16,17,18]. In many coleopteran species, aggregation pheromones are primarily produced by males and released into the environment through specialized glands or excretory products, thereby facilitating conspecific aggregation and host colonization [19,20]. Since the first identification of aggregation pheromones from the hindgut of *Ips paraconfusus* [21], hindgut- and frass-derived volatiles have been increasingly recognized as important sources of semiochemicals in bark beetles and other coleopteran insects. These compounds are predominantly composed of terpenoids and their derivatives, many of which are structurally similar to known pheromone components [22,23]. Therefore, identifying behaviorally active hindgut- and frass-derived volatiles and elucidating the molecular mechanisms underlying their olfactory recognition are essential for understanding insect chemical communication and may provide novel targets for environmentally sustainable pest management.

*Tomicus pilifer* (Coleoptera: Curculionidae: Scolytinae) is an important bark beetle pest of *Pinus koraiensis* and other coniferous trees in northeastern Asia [7,24]. Larvae develop within the phloem of weakened trees, whereas adults subsequently migrate to and feed on healthy shoots, causing twig dieback and substantial forest damage [7,25]. Despite its ecological and economic importance, the molecular mechanisms underlying the recognition of hindgut- and frass-derived semiochemicals in *T. pilifer* remain poorly understood. In the present study, volatile compounds from the hindguts of male and female adults were characterized using gas chromatography–mass spectrometry (GC–MS), and shared behaviorally active compounds were identified through behavioral and electrophysiological assays. Candidate odorant-binding proteins (TpilOBPs) involved in odor recognition were subsequently screened through transcriptomic analyses and tissue-specific expression profiling. Their interactions with active volatiles were further investigated using molecular docking, recombinant protein expression, and fluorescence competitive binding assays, complemented by sequence analyses and structural modeling. This study elucidates the molecular basis of hindgut- and frass-derived semiochemical recognition in *T. pilifer* and provides potential molecular targets for the development of semiochemical-based management strategies against this pest.

## 2. Materials and Methods

### 2.1. Insects

*Tomicus pilifer* adults were collected from infested *Pinus koraiensis* at Hongwei Forest Farm, Qitaihe City, Heilongjiang Province, China (45.4616° N, 131.0011° E). Infested logs were transported to the laboratory and maintained in mesh-covered rearing cages at 25 ± 2 °C, 75 ± 5% relative humidity (RH), and a photoperiod of 14 h light:10 h dark (14 L:10 D) until adult emergence. Newly emerged adults were collected and transferred onto fresh *P. koraiensis* twigs for maturation feeding (approximately 3–5 days). After the adults had completed post-emergence maturation, healthy individuals were selected and used for subsequent experiments.

### 2.2. Extraction of Hindgut and Frass Volatiles

Adult *T. pilifer* obtained from laboratory-reared logs were identified and sexed under a stereomicroscope (Olympus, SZX16, Tokyo, Japan) according to external morphological characteristics. Hindguts were dissected from adults and immediately immersed in pre-chilled n-hexane (Aladdin, Shanghai, China). For each biological replicate, 50 hindguts from either males or females were pooled in a 2 mL amber glass vial containing 1.5 mL n-hexane. Frass produced by male and female adults was collected separately and extracted with pre-chilled n-hexane at a ratio of 0.1 g frass per mL solvent. Samples were incubated in the dark at 4 °C for 24 h. The resulting extracts were filtered through a 0.22 μm organic membrane filter and concentrated under a gentle stream of nitrogen to a final volume of 1 mL. All extracts were stored at −20 °C until subsequent analyses. Three independent biological replicates were prepared for female hindguts, male hindguts, female frass, and male frass, respectively.

### 2.3. GC–MS Analysis and Compound Identification

Volatile extracts were analyzed using a gas chromatography–mass spectrometry (GC–MS) system (7890B GC coupled with 5977A MSD, Agilent Technologies, Santa Clara, CA, USA) equipped with an HP-5MS (30 m × 0.25 mm × 0.25 μm; Agilent Technologies, Santa Clara, CA, USA) capillary column. Helium was used as the carrier gas at a constant flow rate of 1.0 mL min^−1^. Samples (1 μL) were injected in splitless mode, and the injector temperature was maintained at 250 °C. The oven temperature program was as follows: initial temperature of 40 °C held for 2 min, increased to 120 °C at 5 °C min^−1^, then raised to 250 °C at 10 °C min^−1^ and held for 3 min. Mass spectra were acquired using an electron ionization (EI) source operated at 70 eV, with a scanning range of *m*/*z* 40–450. Volatile compounds were tentatively identified by comparison of their mass spectra with those in the NIST17 mass spectral library. The identities of the major monoterpenes were further confirmed by comparison with authentic chemical standards purchased from commercial suppliers (Table S2). The relative abundance of each compound was calculated using the peak area normalization method. Compounds detected in both hindgut and frass extracts of male and female adults were selected as candidate semiochemicals for subsequent behavioral, electrophysiological, and molecular analyses.

### 2.4. Electroantennography (EAG) Recordings

Electroantennographic responses of *T. pilifer* adults to candidate terpenoid compounds were measured using a four-channel EAG system (IDAC-4, Syntech, Buchenbach, Germany). Test compounds were dissolved in n-hexane and serially diluted to concentrations of 0.001, 0.01, 0.1, 1, and 10 μg μL^−1^. Aliquots (10 μL) of each solution were loaded onto filter paper strips (0.5 cm × 3 cm) and placed inside Pasteur pipettes to serve as odor cartridges, whereas n-hexane alone was used as the solvent control. Odor stimuli were delivered as 200 ms pulses at 10 s intervals. Each antenna was exposed sequentially to all concentrations of a given compound, and six independent biological replicates were tested for each treatment. The EAG amplitude (mV) induced by each stimulus was recorded and used as an indicator of antennal responsiveness to the tested volatiles.

### 2.5. Y-Tube Olfactometer Bioassays

Behavioral responses of *T. pilifer* adults to candidate terpenoid compounds were assessed using a Y-tube glass olfactometer (3 cm i.d., 20 cm stem length, 45° arm angle; XLM3-300, Nanjing Xuelai Biotechnology Co., Ltd., Nanjing, China). Purified air was delivered through each arm at a flow rate of 500 mL min^−1^. Aliquots (10 μL) of test solutions (0.01–10 μg μL^−1^ in n-hexane) were applied to filter paper strips and placed in one arm of the olfactometer, while an equal volume of n-hexane was applied to the opposite arm as a control. Individual beetles were released at the base of the stem and allowed to respond to the odor stimuli. Each beetle was allowed a maximum of 15 min to make a choice. An insect was considered to have made a choice when it moved to within 5 cm of either odor source. Individuals that failed to make a choice within 15 min were recorded as non-responders and excluded from the behavioral analysis. The numbers of beetles choosing the treatment and control arms were recorded. Behavioral response (%) was calculated using the following formula:

Behavioral response (%) = Nt/(Nt + Nc) × 100

where Nt is the number of beetles choosing the treatment arm and Nc is the number choosing the control arm.

### 2.6. Screening, Cloning, and Expression Analysis of Candidate OBPs

Transcriptome sequencing was performed using antennae, heads, thoraces, abdomens, legs, and wings collected from female and male adults of *T. pilifer*. For each tissue type, tissues from 50 female adults and 50 male adults were collected separately for each biological replicate. cDNA libraries were constructed by Biomarker Technologies Co., Ltd. (Beijing, China) and sequenced on the Illumina HiSeq platform(Illumina Inc., San Diego, CA, USA). After quality control, clean reads were de novo assembled using Trinity (v2.15), and the resulting transcripts were annotated against the Nr (https://www.ncbi.nlm.nih.gov/), Swiss-Prot (https://www.uniprot.org/), Pfam (https://pfam.xfam.org/), Gene Ontology (GO; http://geneontology.org/), and Kyoto Encyclopedia of Genes and Genomes (KEGG; https://www.genome.jp/kegg/) databases (all accessed on 26 July 2026). Candidate odorant-binding protein (OBP) genes were identified based on annotation results and sequence characteristics. Gene expression levels in different tissues were quantified using Salmon (v1.9.0) and normalized as transcripts per million (TPM). To identify candidate OBPs for functional characterization, phylogenetic analyses were conducted using OBPs identified from *T. pilifer* and functionally characterized OBPs from other coleopteran species. Amino acid sequences were aligned using the MUSCLE algorithm implemented in MEGA7 (v7.0). A neighbor-joining (NJ) phylogenetic tree was constructed with 1000 bootstrap replicates. Candidate OBPs were selected for further functional characterization based on their phylogenetic relationships with functionally characterized coleopteran OBPs, tissue-specific expression patterns, and preliminary molecular docking analyses. The expression profiles of candidate OBP genes in different tissues were further examined by real-time quantitative PCR (RT-qPCR). For RT-qPCR, each biological replicate consisted of tissues pooled from 50 adults. The ribosomal protein L18 (*RPL18*) gene, identified as a stably expressed reference gene through transcriptome analysis, was selected as the reference gene and used as the internal control for RT-qPCR normalization (Table S4). A stably expressed reference gene identified from the transcriptome dataset was used as the internal control. Relative transcript abundance was calculated using the 2^−ΔΔCt^ method. Three biological replicates and three technical replicates were included for each sample.

### 2.7. Sequence Analysis, Structural Prediction, and Molecular Docking of Candidate OBPs

#### 2.7.1. Sequence Analysis of Candidate OBPs

Three candidate odorant-binding proteins (OBPs), *TpilOBP5*, *TpilOBP16*, and *TpilOBP29*, identified from the antennal transcriptome, were selected for bioinformatic analyses. The nucleotide sequences of *TpilOBP5*, *TpilOBP16*, and *TpilOBP29* have been deposited in the NCBI GenBank database under accession numbers PZ717158, PZ717159, and PZ717160, respectively. Open reading frames (ORFs) were predicted using the NCBI ORF Finder (National Center for Biotechnology Information, Bethesda, MD, USA; https://www.ncbi.nlm.nih.gov/orffinder/ (accessed on 7 July 2025)). Theoretical molecular weight (MW) and isoelectric point (pI) were calculated using the ExPASy (Swiss Institute of Bioinformatics, Lausanne, Switzerland; https://web.expasy.org/protparam/; (accessed on 13 July 2025)) ProtParam tool. Signal peptides were predicted using SignalP 6.0 (https://services.healthtech.dtu.dk/services/SignalP/ (accessed on 13 July 2025)). Multiple sequence alignments were performed using the MUSCLE algorithm implemented in MEGA 7. Phylogenetic relationships were inferred using the neighbor-joining (NJ) method with 1000 bootstrap replicates. The resulting phylogenetic tree was visualized using Interactive Tree Of Life (iTOL) (EMBL, Heidelberg, Germany; https://itol.embl.de/ (accessed on 10 August 2025)). Conserved motifs were identified using the MEME Suite (https://meme-suite.org/) online server.

#### 2.7.2. Structural Prediction of Candidate OBPs

Three-dimensional structures of TpilOBP5, TpilOBP16, and TpilOBP29 were predicted using the SWISS-MODEL server(University of Basel, Basel, Switzerland; https://swissmodel.expasy.org/ (accessed on 18 December 2025)). Suitable template structures were selected based on sequence similarity to the target proteins. Model quality was evaluated using the Global Model Quality Estimation (GMQE) score provided by SWISS-MODEL. The stereochemical quality of the predicted structures was further assessed by Ramachandran plot analysis using the PROCHECK implemented in the SAVES server (https://saves.mbi.ucla.edu/; accessed on 29 December 2025).

#### 2.7.3. Molecular Docking Analysis

Candidate ligands were obtained from the PubChem database(National Center for Biotechnology Information, Bethesda, MD, USA; https://pubchem.ncbi.nlm.nih.gov/ (accessed on 8 July 2025)). Ligand selection was based on volatile compounds identified by GC–MS (Table S1) together with compounds previously reported in *T. pilifer* and related studies [26], with emphasis on volatile compounds potentially involved in host recognition and chemical communication. Protein and ligand structures were prepared using AutoDock Tools (ADT, v 1.5.7) by adding polar hydrogen atoms and Gasteiger charges. Ligands were subjected to energy minimization prior to docking. Molecular docking analyses were performed using AutoDock Vina (v 1.2.3) to evaluate the potential interactions between candidate OBPs and volatile compounds associated with host location and chemical communication. The docking results were used as one of the criteria for selecting candidate OBPs for subsequent functional characterization. Protein–ligand binding conformations were visualized using PyMOL (v 2.5.5), and interacting residues and interaction types were analyzed using Discovery Studio 2019.

### 2.8. RNA Extraction, cDNA Synthesis, and Gene Cloning

Total RNA was extracted from the antennae of adult *T. pilifer* using an RNA extraction kit (Beyotime, Shanghai, China) according to the manufacturer’s instructions. RNA integrity and concentration were assessed by 1% agarose gel electrophoresis and spectrophotometric analysis. First-strand cDNA was synthesized using a reverse transcription kit following the manufacturer’s protocol. Gene-specific primers were designed based on the OBP gene sequences obtained from the antennal transcriptome. PCR amplification was performed using cDNA as the template. The amplified products were purified and ligated into a cloning vector using a homologous recombination method, followed by transformation into Trans-T1 competent cells (TransGen Biotech, Beijing, China). Positive transformants were selected on antibiotic-containing medium and verified by colony PCR. Confirmed positive clones were sequenced by Sangon Biotech Co., Ltd. (Shanghai, China) to obtain the complete open reading frame (ORF) sequences of the target genes.

### 2.9. Expression and Purification of Recombinant Proteins

The coding sequences of *TpilOBP5*, *TpilOBP16*, and *TpilOBP29* were cloned into prokaryotic expression vectors following sequence verification. *TpilOBP5* was inserted into the pET-32b expression vector. Preliminary expression tests indicated that recombinant *TpilOBP16* and *TpilOBP29* were mainly expressed as inclusion bodies without MBP fusion. Therefore, these two proteins were cloned into maltose-binding protein (MBP) fusion expression vectors to enhance their soluble expression. The MBP fusion vectors used for *TpilOBP16* and *TpilOBP29* expression contained a His-tag, allowing purification of recombinant proteins using Ni-NTA affinity chromatography. The resulting recombinant plasmids were transformed into Escherichia coli BL21 (DE3) competent cells for heterologous expression. Transformed cells were cultured in LB medium supplemented with 100 μg/mL ampicillin at 37 °C with shaking until the optical density at 600 nm (OD600) reached 0.6–0.8. Protein expression was induced by the addition of isopropyl β-D-1-thiogalactopyranoside (IPTG), followed by incubation at 16 °C and 160 rpm for 24 h. Bacterial cells were harvested by centrifugation and resuspended in 20 mmol/L Tris-HCl buffer. The cells were disrupted by ultrasonication and subsequently centrifuged to separate the soluble and insoluble fractions. Protein expression was analyzed by sodium dodecyl sulfate–polyacrylamide gel electrophoresis (SDS-PAGE). Recombinant fusion proteins were purified using Ni-NTA affinity chromatography. Target proteins were eluted with an imidazole gradient and subsequently desalted and concentrated using ultrafiltration centrifugal devices. The purified fusion proteins of TpilOBP5, TpilOBP16, and TpilOBP29 were subsequently used for fluorescence competitive binding assays and protein–ligand interaction analyses.

### 2.10. Fluorescence Competitive Binding Assays

The binding affinities of TpilOBP5, TpilOBP16, and TpilOBP29 toward volatile compounds were evaluated using fluorescence competitive binding assays. Test ligands included the five major hindgut- and frass-derived volatile compounds identified by GC–MS analysis, together with additional candidate ligands selected based on molecular docking analyses. Fluorescence measurements were performed using a Varioskan LUX multimode microplate reader (Thermo Fisher Scientific, Waltham, MA, USA) at 27 ± 1 °C. N-phenyl-1-naphthylamine (1-NPN; Aladdin, Shanghai, China) was used as the fluorescent probe. Structural studies have shown that 1-NPN binds within the canonical hydrophobic ligand-binding pocket of insect OBPs and can be competitively displaced by odorant ligands, making it a widely used fluorescent reporter in competitive binding assays [26]. Fluorescence emission spectra were recorded from 350 to 500 nm with an excitation wavelength of 337 nm. To determine the dissociation constant (Kd) of the protein–probe complex, purified recombinant proteins (2 μM in 50 mM Tris–HCl (Yeasen, Shanghai, China) buffer, pH 7.4) were titrated with increasing concentrations of 1-NPN (2–20 μM). Competitive binding assays were subsequently performed by adding increasing concentrations of individual ligands (0–25 μM) to the protein–1-NPN complex. All measurements were conducted in triplicate. Binding parameters were estimated using Scatchard analysis. The inhibition constant (Ki) of each ligand was calculated according to the Cheng–Prusoff equation, as previously described [26,27].

### 2.11. Statistical Analysis

All quantitative data are presented as mean ± standard error (SE). Statistical analyses of RT-qPCR, electroantennography (EAG), and fluorescence binding assay data were performed using SPSS 17.0 (SPSS Inc., Chicago, IL, USA). Differences among multiple treatments were evaluated by one-way analysis of variance (ANOVA) followed by Tukey’s honestly significant difference (HSD) test for multiple comparisons. Behavioral responses in Y-tube olfactometer assays were analyzed using Student’s *t*-test. Differences were considered statistically significant at *p* < 0.05. For fluorescence competitive binding assays, half-maximal inhibitory concentration (IC_50_) values were estimated from competitive binding curves, and inhibition constants (Ki) were calculated using Origin 2022 (OriginLab Corporation, Northampton, MA, USA) according to the Cheng–Prusoff equation. Graph preparation and data visualization were performed using GraphPad Prism 9.0 (GraphPad Software, Boston, MA, USA).

## 3. Results

### 3.1. Identification of Common Hindgut- and Frass-Derived Volatile Compounds in Tomicus pilifer

GC–MS analysis identified 43 and 54 volatile compounds from the hindguts of female and male *Tomicus pilifer* adults, respectively, whereas 54 and 56 volatile compounds were detected from female and male frass samples, respectively (Table S1). These compounds mainly belonged to terpenes, alkanes, esters, ethers, alcohols, and benzene derivatives, as well as a small number of nitriles and nitrogen-containing heterocyclic compounds.

A total of eight volatile compounds were consistently detected in both hindgut and frass samples of male and female adults (Table 1). These included five monoterpenes—namely, α-pinene, camphene, β-myrcene, 3-carene, and D-limonene—together with three straight-chain alkanes: pentacosane, hexadecane, and octacosane. In general, the relative abundances of these compounds were higher in frass than in hindgut extracts. Therefore, the eight common volatile compounds were selected as candidate semiochemicals for subsequent electrophysiological, behavioral, and molecular analyses.

### 3.2. Electrophysiological and Behavioral Responses of T. pilifer to Candidate Terpenoid Compounds

#### 3.2.1. Electroantennography (EAG) Responses

**Figure 1:**
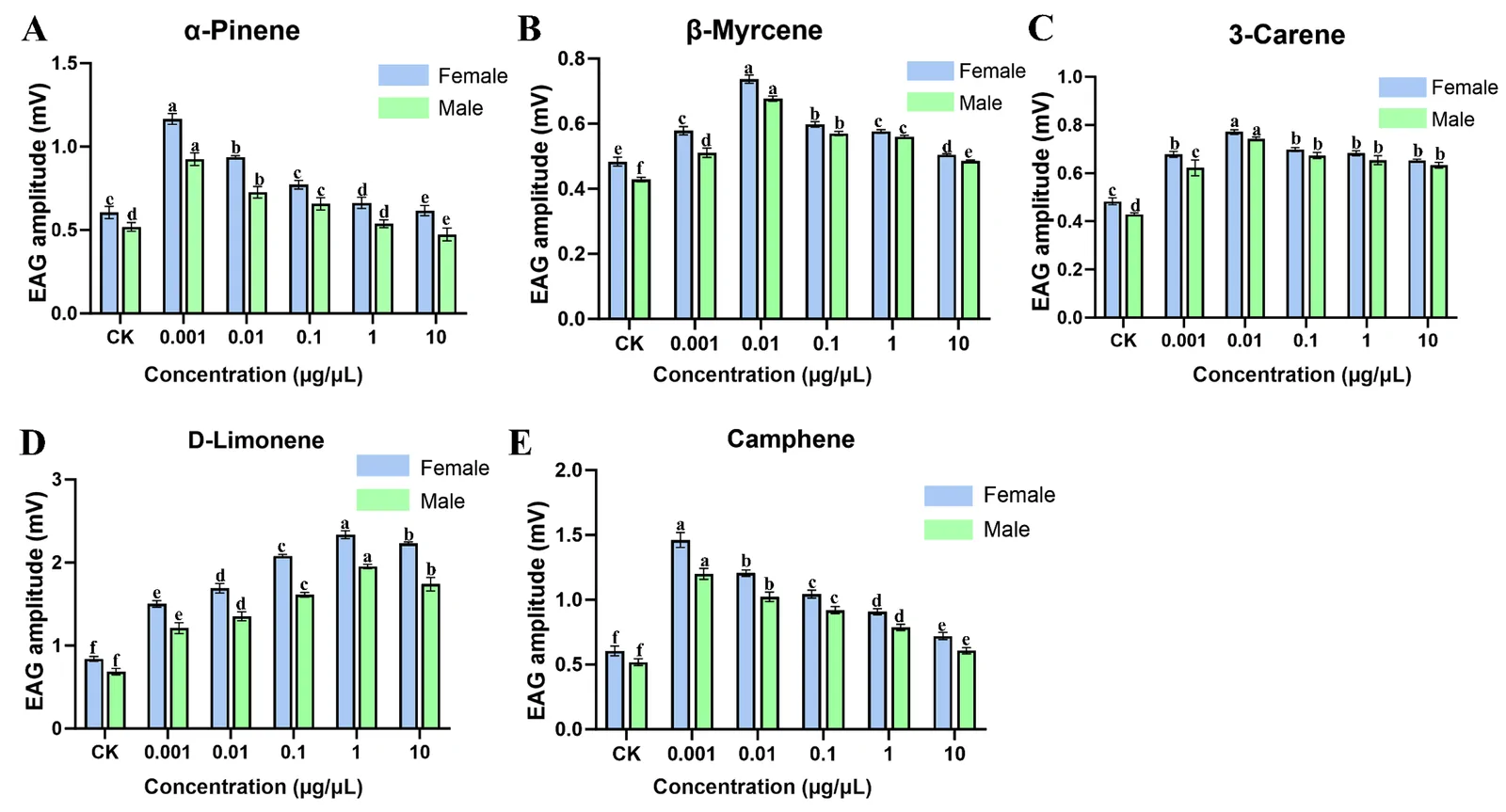
Electroantennographic (EAG) responses of male and female *T. pilifer* adults to five common volatile compounds identified from hindgut and frass samples at different concentrations (0.001–10 μg/μL). (**A**–**E**) represent the EAG responses to α-pinene, β-myrcene, 3-carene, D-limonene, and camphene, respectively.

The EAG assays revealed that five of the eight volatile compounds commonly detected in hindgut and frass samples elicited significant antennal responses in both male and female *T. pilifer* adults, including α-pinene, camphene, β-myrcene, 3-carene, and D-limonene (Figure 1). All electrophysiologically active compounds belonged to the monoterpene class. Overall, male and female adults exhibited similar response patterns to the five active compounds, and the EAG responses induced by all treatments were significantly higher than those elicited by the solvent control. Significant differences in response intensity were observed among compounds. D-limonene elicited the strongest antennal response, with response amplitudes increasing as concentration increased and reaching the highest value within the tested concentration range at 1 μg/μL, followed by a decrease at 10 μg/μL. In contrast, camphene induced a relatively high EAG response at the lowest concentration tested (0.001 μg/μL), followed by a gradual decline with increasing concentration. α-Pinene consistently elicited relatively high antennal responses across concentrations, whereas β-myrcene and 3-carene induced comparatively lower response amplitudes.

#### 3.2.2. Y-Tube Olfactometer Assays

**Figure 2:**
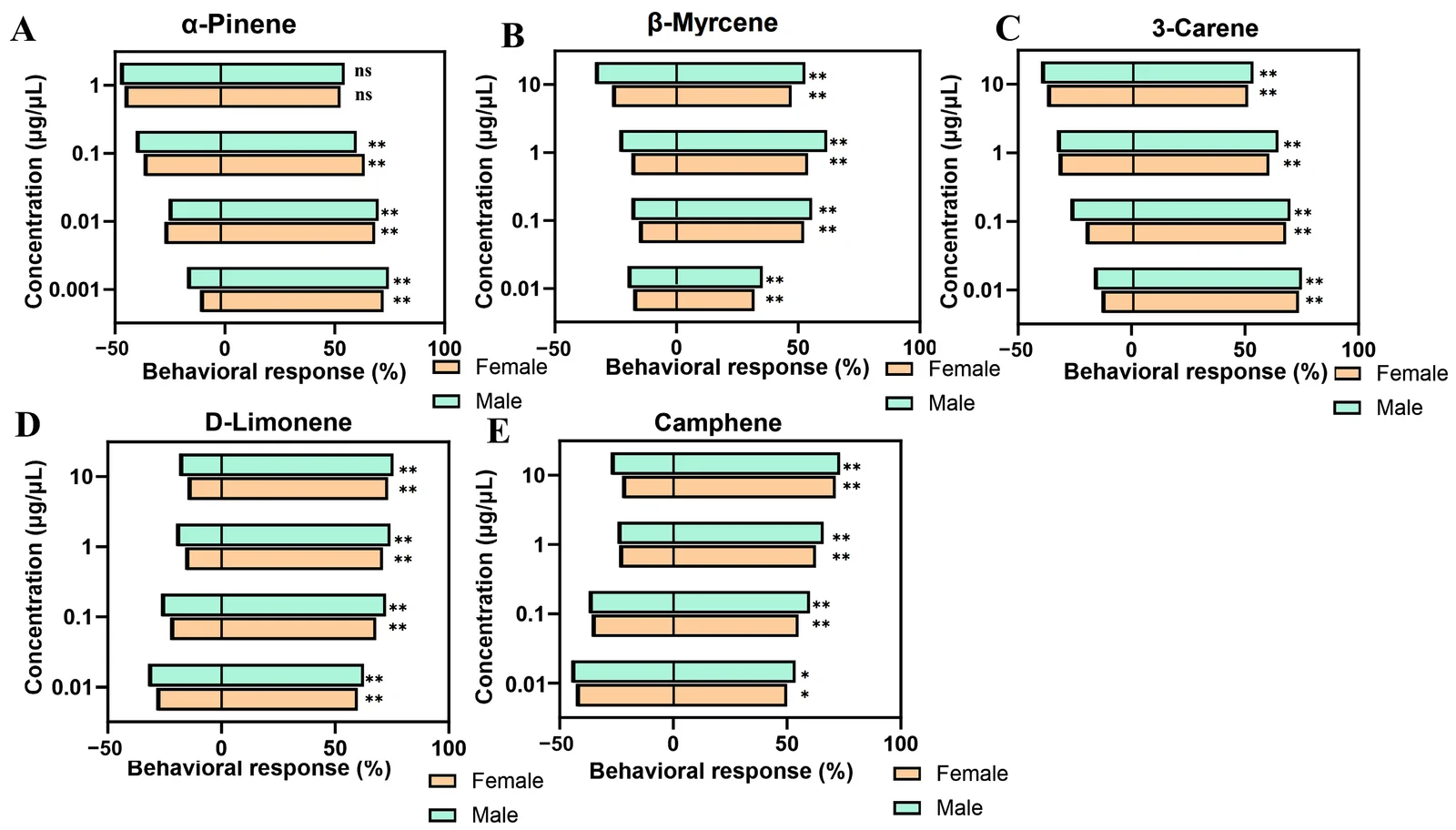
Y-tube olfactometer responses of *T. pilifer* adults to hindgut-derived monoterpenes. The vertical axis at 0 represents no preference. Values on the left side of the axis indicate responses toward the hexane control, whereas values on the right side indicate responses toward the odor treatment. (**A**–**E**) represent responses to α-pinene, β-myrcene, 3-carene, D-limonene, and camphene, respectively. Asterisks indicate significant differences determined by Student’s *t*-test (* *p* < 0.05; ** *p* < 0.01; ns, not significant).

The Y-tube olfactometer assays showed that all five monoterpenes elicited behavioral responses in male and female *T. pilifer* adults over the tested concentration range (0.001–10 μg/μL), although response patterns varied among compounds (Figure 2). 3-Carene, D-limonene, and camphene exhibited significant attraction at all tested concentrations. α-Pinene elicited significant attraction at concentrations ranging from 0.001 to 0.1 μg/μL, whereas no significant behavioral response was observed at 1 μg/μL (*p* > 0.05). β-Myrcene significantly attracted both male and female adults at all tested concentrations and produced the highest behavioral response index at 1 μg/μL. Within the concentration range tested, none of the five monoterpenes induced significant repellency toward *T. pilifer* adults. In addition, male and female adults exhibited generally similar behavioral responses to all tested compounds.

### 3.3. Identification and Expression Profiling of Candidate OBPs

#### 3.3.1. Identification of Candidate OBPs

A total of 51 OBP genes were identified from the antennal transcriptome of *T. pilifer*. Candidate OBPs were screened based on phylogenetic relationships with functionally characterized coleopteran OBPs and preliminary molecular docking analyses. Phylogenetic analysis showed that *TpilOBP5*, *TpilOBP16*, and *TpilOBP29* clustered with the functionally characterized *PparOBP1*, *TyunOBP3*, and *TyunOBP2*, respectively (Figure S1), suggesting their potential involvement in odor recognition. In addition, preliminary molecular docking analyses suggested that all three candidate OBPs possessed favorable predicted binding affinities toward behaviorally active volatile compounds. Therefore, *TpilOBP5*, *TpilOBP16*, and *TpilOBP29* were selected for subsequent functional characterization.

#### 3.3.2. Transcriptome-Based Expression Profiles of Candidate OBPs

**Figure 3:**
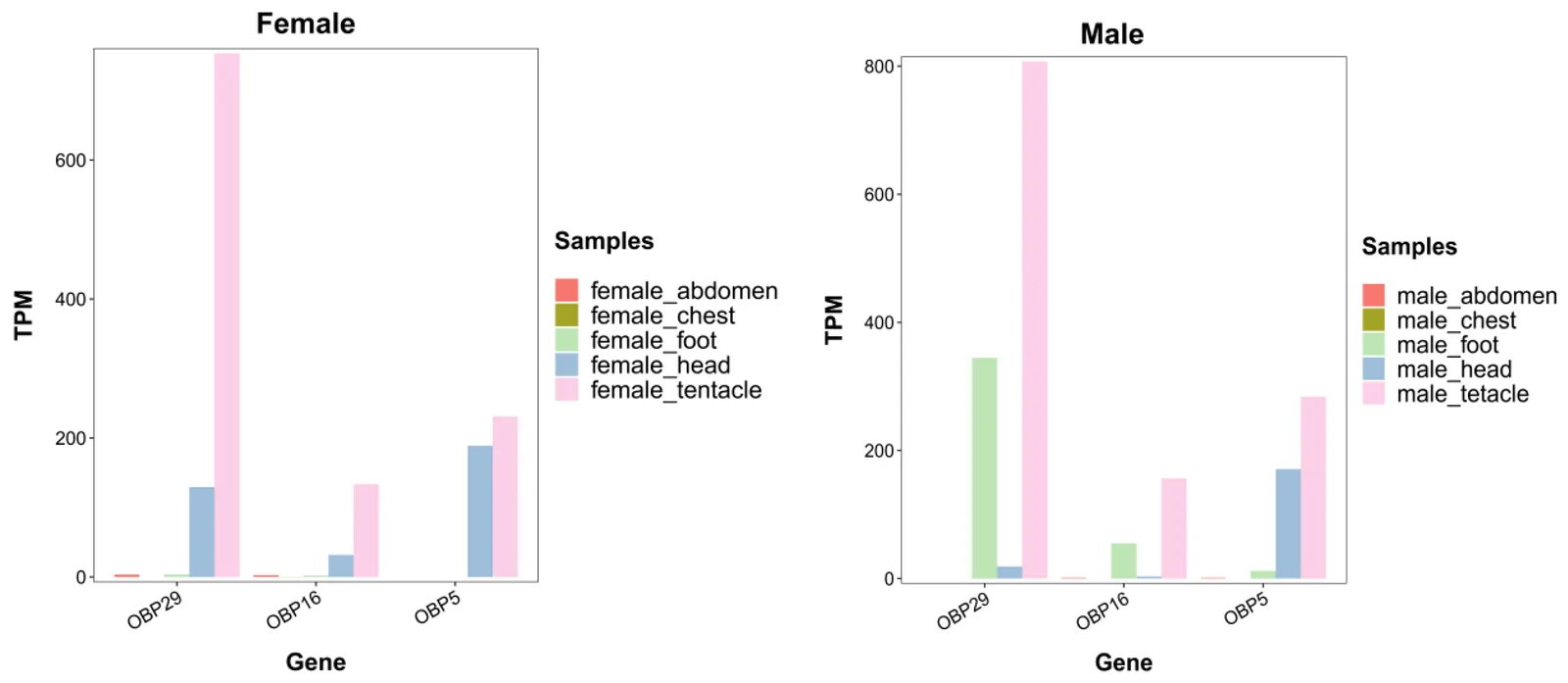
Tissue-specific transcriptome expression profiles of *TpilOBP5*, *TpilOBP16*, and *TpilOBP29* in male and female *T. pilifer* adults.

Transcriptome expression analyses revealed that *TpilOBP5*, *TpilOBP16*, and *TpilOBP29* were predominantly expressed in the antennae of both female and male adults (Figure 3). Among them, *TpilOBP29* showed substantially higher transcript abundance in the antennae than in any other tissue and displayed a pronounced antenna-enriched expression pattern. Similarly, *TpilOBP16* and *TpilOBP5* were primarily expressed in the antennae, with relatively low expression detected in the head and negligible expression in the thorax, abdomen, legs, and wings. Overall, the tissue expression patterns were similar between females and males. A low level of *TpilOBP29* expression was detected in the legs of male adults; however, its transcript abundance remained markedly higher in the antennae than in any other tissue.

#### 3.3.3. Tissue-Specific Expression Profiles of Candidate OBPs

**Figure 4:**
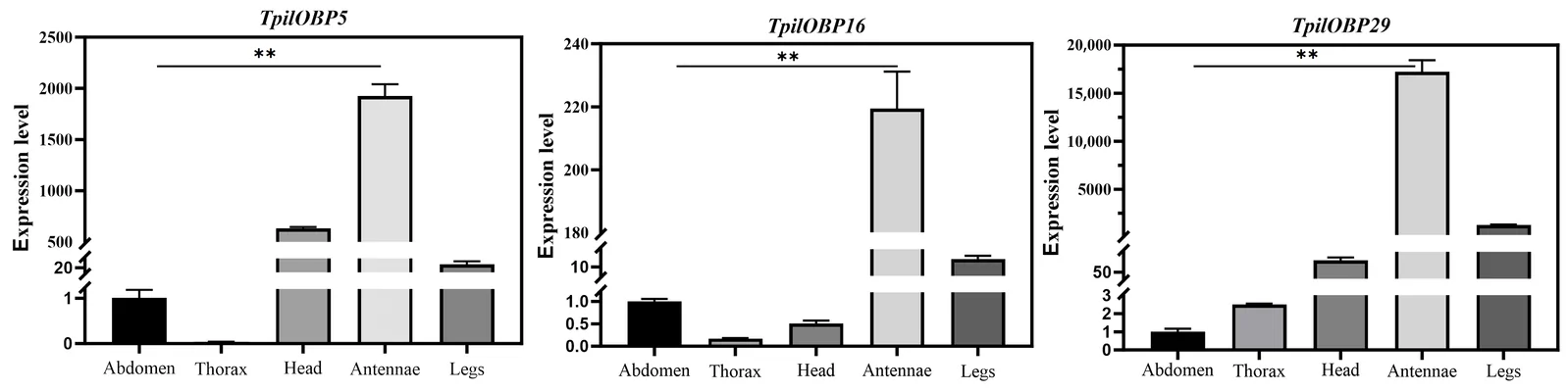
Tissue-specific expression profiles of *TpilOBP5*, *TpilOBP16*, and *TpilOBP29* in adult *T. pilifer.* Gene expression levels in different tissues were determined by RT-qPCR. Relative expression levels were calculated using the 2^−ΔΔCt^ method with the abdomen as the calibrator tissue. Data are presented as mean ± SE (*n*` = 3). Asterisks indicate significant differences between antennae and the calibrator tissue (** *p* < 0.01).

The tissue-specific expression patterns of *TpilOBP5*, *TpilOBP16*, and *TpilOBP29* were further examined by RT-qPCR. All three genes exhibited significantly higher expression levels in the antennae than in other tissues, displaying pronounced antenna-enriched expression patterns (Figure 4). The overall expression trends obtained by RT-qPCR were consistent with those observed in the transcriptome dataset. Among the three candidate genes, *TpilOBP5* showed its highest expression level in the antennae, with relatively lower expression detected in the head and legs and minimal expression in the thorax. Similarly, *TpilOBP16* was predominantly expressed in the antennae, whereas its transcript abundance remained comparatively low in the head and legs. In contrast, *TpilOBP29* exhibited the strongest antenna-enriched expression pattern, with transcript levels in the antennae substantially exceeding those in all other tissues. Only low levels of expression were detected in the head and legs.

Overall, *TpilOBP5*, *TpilOBP16*, and *TpilOBP29* were predominantly expressed in the antennae, whereas expression in the thorax, abdomen, wings, and legs remained comparatively low.

### 3.4. Sequence Characterization and Phylogenetic Analyses of Candidate OBPs

#### 3.4.1. Physicochemical Properties and Phylogenetic Relationships

The full-length open reading frames (ORFs) of *TpilOBP5*, *TpilOBP16*, and *TpilOBP29* were 426, 456, and 399 bp, encoding 141, 151, and 134 amino acids, respectively. The predicted molecular weights of the mature proteins were 15.5, 16.6, and 14.7 kDa, respectively, whereas the theoretical isoelectric points (pI) after removal of the signal peptides were 5.16, 5.30, and 5.21.

**Figure 5:**
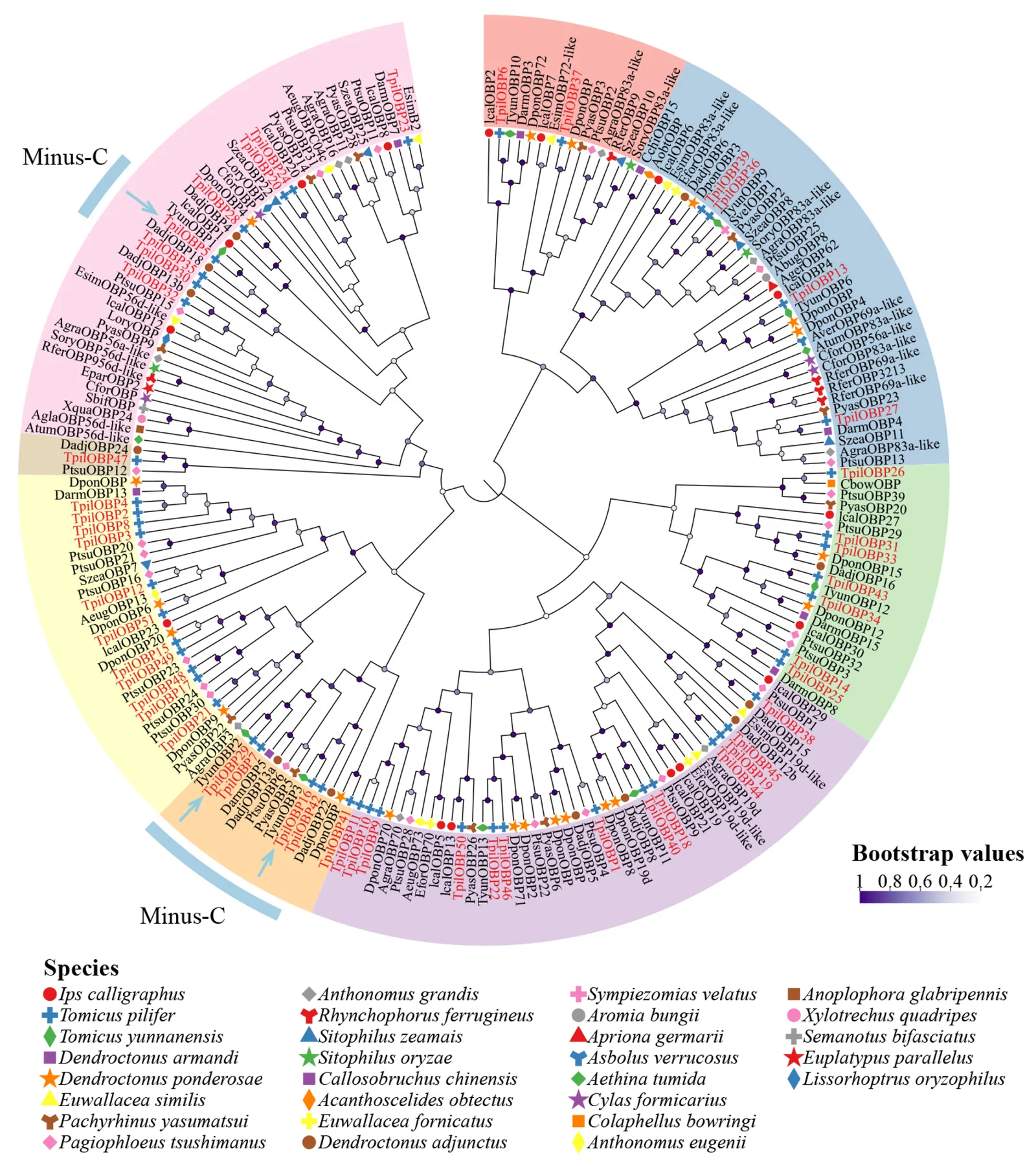
Phylogenetic relationships of odorant-binding proteins (OBPs) from *T. pilifer* and other coleopteran species. The red font indicates OBP genes identified from the antennal transcriptome of *T. pilifer*, and arrows indicate the three candidate OBPs selected for this study (TpilOBP5, TpilOBP16, and TpilOBP29).

A phylogenetic analysis was conducted using OBP sequences from *T. pilifer* and 31 coleopteran species, including *Ips calligraphus*, *Tomicus yunnanensis*, *Dendroctonus armandi*, *Pachyrhinus yasumatsui*, and *Aromia bungii* (Figure 5). The identified *T. pilifer* OBPs were distributed across multiple OBP subfamilies. Most OBPs clustered with homologous sequences from bark beetles and other coleopteran species. *TpilOBP5*, *TpilOBP16*, and *TpilOBP29* were all assigned to the Minus-C OBP subfamily and clustered with homologous OBPs from related scolytine species, forming well-supported clades.

#### 3.4.2. Conserved Motif Analyses of Candidate OBPs

**Figure 6:**
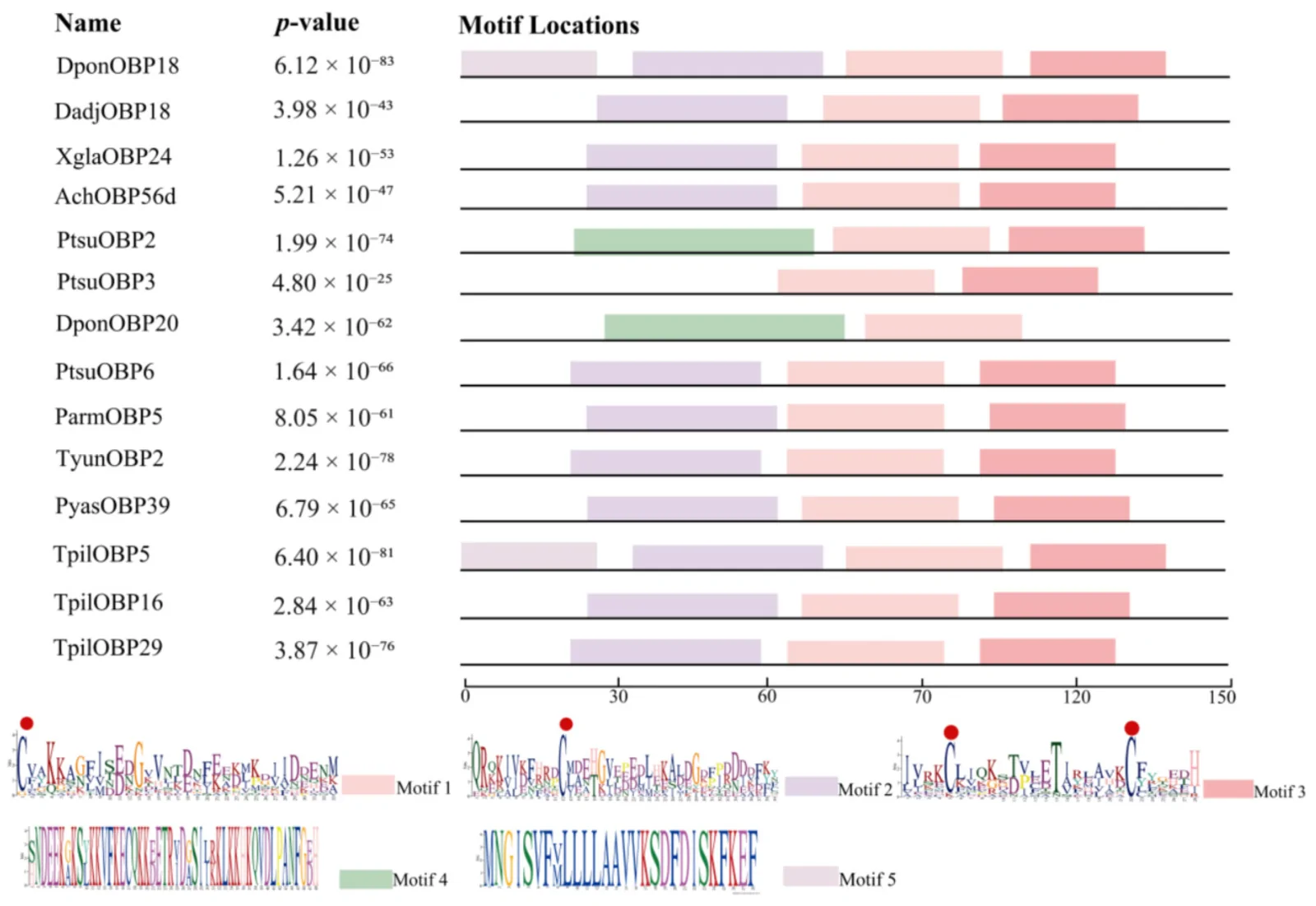
Conserved motif composition of candidate OBPs from *T. pilifer* and their homologous proteins. Colored boxes represent different conserved motifs (Motifs 1–5) and their relative positions within each protein sequence. Protein names and motif-matching significance values (*p*-values) are shown on the left. The lower panel displays the amino acid sequence logos of the five identified motifs. Conserved cysteine residues within the motifs are indicated by red dots.

A total of 14 OBP sequences, including *TpilOBP5*, *TpilOBP16*, and *TpilOBP29* from *T. pilifer* and 11 homologous OBPs from closely related species, were subjected to conserved motif analysis. As shown in Figure 6, four to five conserved motifs (Motifs 1–5) were identified among the analyzed sequences. The predominant motif arrangement was Motif 2–Motif 1–Motif 3, whereas some sequences additionally contained Motif 4 and/or Motif 5. Motifs 1–3 were detected in all analyzed OBPs, whereas Motifs 4 and 5 were present only in a subset of sequences. Overall, the motif composition and arrangement were highly similar among the three candidate OBPs and their homologs.

#### 3.4.3. Structural Modeling of Candidate OBPs

**Figure 7:**
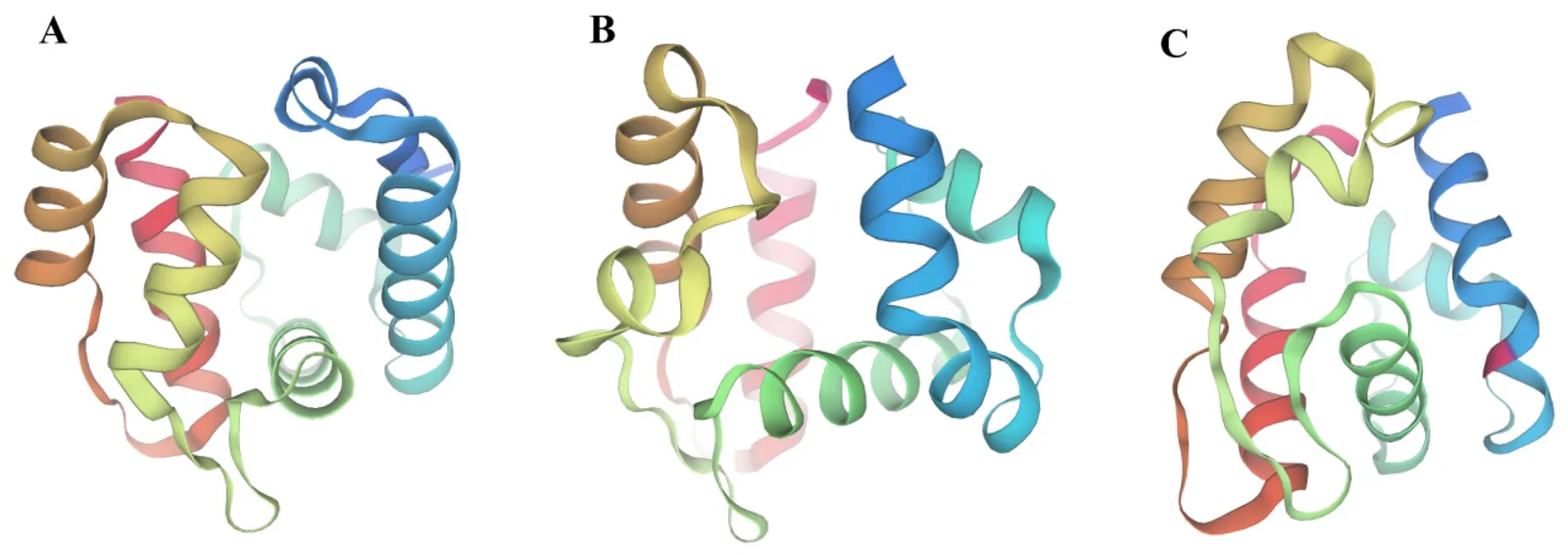
Three-dimensional homology models of TpilOBP5, TpilOBP16, and TpilOBP29 from *T. pilifer*. (**A**) TpilOBP5; (**B**) TpilOBP16; (**C**) TpilOBP29. Protein structures were generated by homology modeling based on homologous odorant-binding proteins from *Tomicus yunnanensis* using SWISS-MODEL. Different colors were automatically assigned for visualization of the predicted structures and do not represent conserved motifs.

To investigate the structural characteristics of the candidate OBPs, three-dimensional homology models were generated for TpilOBP5, TpilOBP16, and TpilOBP29 (Figure 7). All three models were constructed using a homologous OBP from *Tomicus yunnanensis* as the template. The sequence identities between the target proteins and the selected template were 91.06%, 91.89%, and 91.96% for TpilOBP5, TpilOBP16, and TpilOBP29, respectively (Figure S2).

The predicted structures exhibited the characteristic architecture of insect OBPs and were predominantly composed of six α-helices connected by loop regions. No extensive β-sheet structures were observed. Distinct internal cavities were present in all three models. TpilOBP29 appeared to possess a more compact overall conformation and a relatively enclosed internal cavity (Figure 7C), whereas TpilOBP5 and TpilOBP16 displayed comparatively more open helical arrangements (Figure 7A,B).

Model quality was evaluated using GMQE scores and Ramachandran plot analyses. The majority of amino acid residues were located within favored regions of the Ramachandran plots, supporting the overall stereochemical quality of the predicted structures (Figure 7).

#### 3.4.4. Molecular Docking Analyses of Candidate OBPs with Volatile Compounds

Molecular docking analyses were conducted between TpilOBP5, TpilOBP16, TpilOBP29, and 21 volatile compounds and the predicted binding energies are summarized in Table 2. Among the three candidate OBPs, TpilOBP29 exhibited the lowest overall predicted binding energies, followed by TpilOBP16 and TpilOBP5. Based on the GC–MS identification, electrophysiological recordings, and behavioral assays, five biologically active monoterpenes (α-pinene, camphene, β-myrcene, 3-carene, and D-limonene) were selected for further analysis. The predicted binding energies between these compounds and the three candidate OBPs ranged from −4.7 to −7.6 kcal/mol (Table 2). Among the tested proteins, TpilOBP29 exhibited the lowest predicted binding energies with α-pinene and camphene (both −7.6 kcal/mol). TpilOBP16 showed its lowest predicted binding energies with 3-carene and D-limonene (both −6.2 kcal/mol), whereas TpilOBP5 displayed comparatively higher binding energies, with its lowest value observed for D-limonene (−5.0 kcal/mol). The five biologically active monoterpenes consistently exhibited more negative predicted binding energies with the three candidate OBPs compared with the other tested volatile compounds (Table S2), indicating stronger predicted interactions with these proteins.

### 3.5. Validation of Ligand-Binding Properties and Interaction Analyses of Candidate OBPs

#### 3.5.1. Expression and Purification of Recombinant OBPs

**Figure 8:**
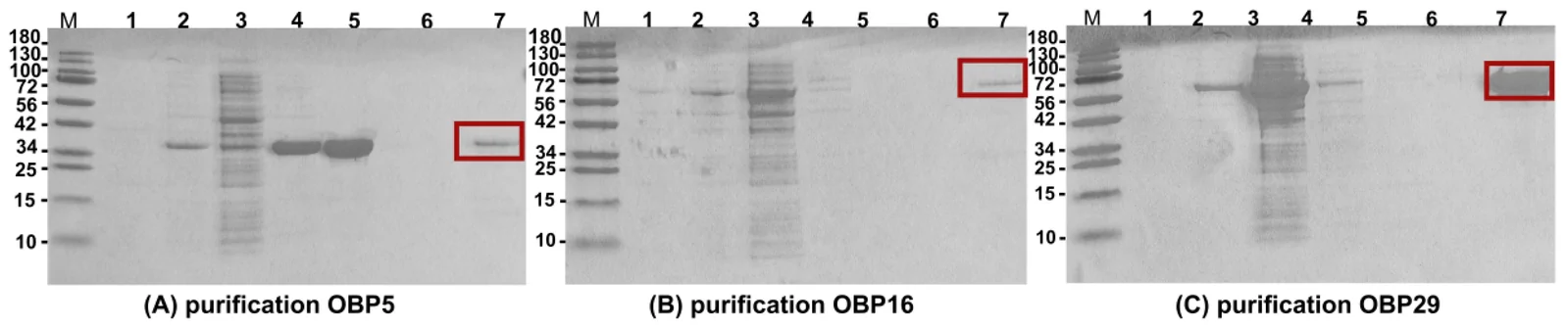
SDS-PAGE analysis of recombinant TpilOBP5, TpilOBP16, and TpilOBP29 during expression and purification. Recombinant proteins were analyzed by 15% SDS-PAGE following IPTG induction and affinity purification. Lane M, protein molecular weight marker (10–180 kDa); Lane 1, soluble fraction before induction; Lane 2, soluble fraction after induction with 0.5 mM IPTG; Lane 3, flow-through fraction; Lane 4, 10 mM imidazole elution fraction; Lane 5, 30 mM imidazole elution fraction; Lane 6, 50 mM imidazole elution fraction; Lane 7, purified recombinant protein. The red boxes indicate the target recombinant protein bands.

Recombinant TpilOBP5, TpilOBP16, and TpilOBP29 were successfully expressed in Escherichia coli and subsequently purified. SDS–PAGE analysis showed that all three recombinant proteins were predominantly detected in the soluble fraction and were successfully recovered after purification (Figure 8). The apparent molecular masses of the recombinant fusion proteins were approximately 32.5, 59.5, and 57.8 kDa for TpilOBP5, TpilOBP16, and TpilOBP29, respectively, which were consistent with the expected sizes of the corresponding expression constructs. The purified proteins appeared as distinct bands on 15% SDS–PAGE gels, indicating successful expression and purification for subsequent binding assays.

#### 3.5.2. Fluorescence Competitive Binding Assays

**Figure 9:**
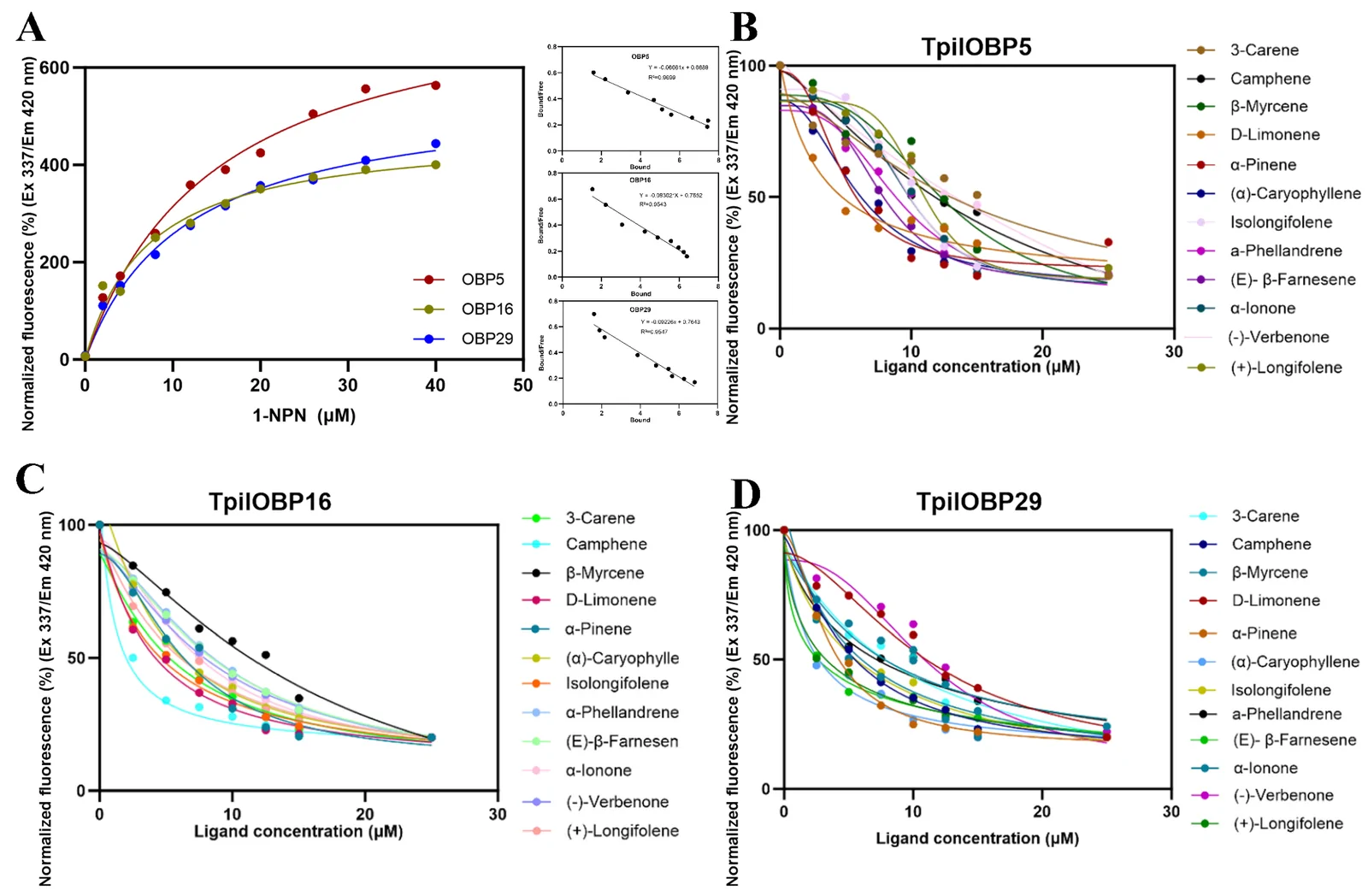
Fluorescence competitive binding assays of recombinant TpilOBP5, TpilOBP16, and TpilOBP29 with selected terpene compounds. (**A**) Binding curves and Scatchard plots of TpilOBP5, TpilOBP16, and TpilOBP29 with the fluorescent probe 1-NPN. (**B**) Competitive binding curves of TpilOBP5 with selected ligands. (**C**) Competitive binding curves of TpilOBP16 with selected ligands. (**D**) Competitive binding curves of TpilOBP29 with selected ligands.

The binding properties of recombinant TpilOBP5, TpilOBP16, and TpilOBP29 toward target volatile compounds and selected high-affinity terpenes identified by molecular docking were evaluated using fluorescence competitive binding assays with 1-NPN as the fluorescent probe (2 μM) (Figure 9). The fluorescence intensities of all three OBPs increased with increasing concentrations of 1-NPN and gradually reached saturation. Scatchard analysis indicated that all three proteins were able to bind 1-NPN, with dissociation constants (Kd) in the micromolar range (Table 3). Among them, TpilOBP16 exhibited the lowest Kd value (7.11 μM) (Figure 9A).

Competitive binding assays revealed differences in ligand-binding affinities among the three candidate OBPs. TpilOBP29 showed relatively low Ki values for α-pinene (3.16 ± 0.18 μM), camphene (4.35 ± 0.24 μM), and β-myrcene (4.30 ± 0.22 μM). For TpilOBP16, the lowest Ki value was observed for camphene (0.96 ± 0.05 μM). TpilOBP5 exhibited the lowest Ki value for D-limonene (2.91 ± 0.14 μM), followed by α-pinene.

Among the non-target terpene compounds, TpilOBP29 showed relatively low Ki values for β-caryophyllene (1.94 ± 0.09 μM) and (E)-β-farnesene (2.65 ± 0.14 μM). TpilOBP16 also exhibited measurable binding affinities toward isolongifolene (3.82 ± 0.21 μM) and β-caryophyllene (4.01 ± 0.22 μM) (Figure 9B–D).

#### 3.5.3. Visualization of Binding Modes Between Candidate OBPs and High-Affinity Ligands

**Figure 10:**
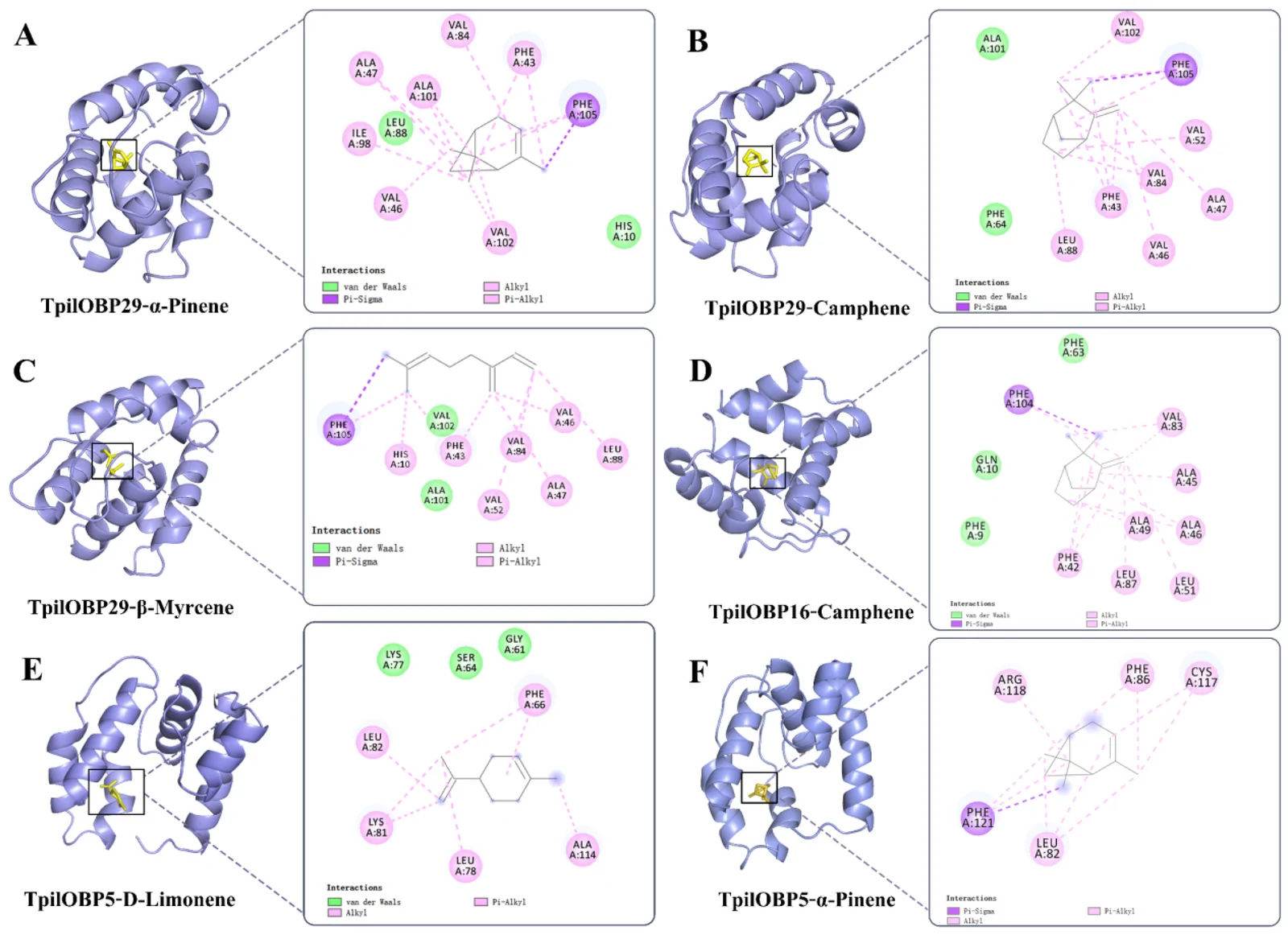
Predicted binding modes and key interacting residues of candidate TpilOBPs with selected terpene ligands based on molecular docking analyses. (**A**) TpilOBP29–α-pinene; (**B**) TpilOBP29–camphene; (**C**) TpilOBP29–β-myrcene; (**D**) TpilOBP16–camphene; (**E**) TpilOBP5–α-pinene; (**F**) TpilOBP5–D-limonene. Amino acid residues involved in ligand interactions are shown within the binding pocket.

To further characterize the interactions between candidate OBPs and high-affinity ligands, three-dimensional models of TpilOBP5, TpilOBP16, and TpilOBP29 were used for molecular docking and visualization analyses with the five target volatile compounds and selected high-affinity terpene compounds identified in the fluorescence competitive binding assays (Figure 10).

Docking visualization showed that the interactions between terpene ligands and the three candidate OBPs were predominantly composed of van der Waals and alkyl interactions. Multiple amino acid residues were involved in ligand binding within the predicted binding cavities of TpilOBP5, TpilOBP16, and TpilOBP29.

For TpilOBP29, α-pinene, camphene, 3-carene, D-limonene, β-myrcene, longifolene, and β-caryophyllene interacted mainly with residues Phe43, Val46, Ala47, Val52, Val84, Leu88, Val102, and Phe105. Additional interactions involving His10, Phe64, Ile72, Val80, and Ala101 were also observed for some ligands. In TpilOBP5, D-limonene interacted primarily with Phe66, Leu62, Lys81, Leu78, and Ala114, whereas longifolene and β-caryophyllene were associated with residues Leu82, Phe86, Cys117, Arg118, and Phe121. For TpilOBP16, interactions with D-limonene and longifolene involved residues Met28, Phe38, Phe42, Phe104, Tyr105, Ile101, and Leu111.

Overall, the docking analyses identified distinct ligand-contacting residues among the three candidate OBPs, with van der Waals and hydrophobic interactions representing the predominant interaction types observed in the predicted complexes.

## 4. Discussion

The present study aimed to identify the major volatile compounds released from the hindgut and frass of *Tomicus pilifer* and to elucidate the roles of key odorant-binding proteins (OBPs) involved in the olfactory recognition of these compounds. As a typical bark beetle, *T. pilifer* relies heavily on chemical cues for host location and intraspecific communication [28]. Among these cues, volatile compounds released from the hindgut and frass have long been considered important semiochemical signals involved in mediating aggregation and behavioral regulation [29]. Although terpenes are primarily derived from host plants, selective accumulation and metabolic transformation during insect feeding can alter both the composition and relative abundance of these compounds, thereby conferring potential semiochemical functions on hindgut- and frass-derived volatiles. Such modifications not only reshape the chemical profile of the emitted odor blend but may also influence ligand recognition and binding by OBPs, ultimately affecting olfactory perception and subsequent behavioral responses [30,31]. However, the molecular relationship between hindgut- and frass-derived volatile compounds and their corresponding OBPs in *T. pilifer* has remained largely unexplored. Therefore, by integrating volatile profiling, behavioral and electrophysiological assays, and molecular functional analyses, the present study investigated how adult *T. pilifer* recognizes specific volatile cues through its peripheral olfactory system and responds to these behaviorally relevant compounds.

Since the 1960s, studies have demonstrated that aggregation pheromones accumulate in the hindgut of male *Ips confusus*, and three biologically active terpene-derived compounds, (−)-2-methyl-6-methylene-7-octen-4-ol, (+)-cis-verbenol, and (+)-2-methyl-6-methylene-2,7-octadien-4-ol, were first identified from male frass [32]. These findings suggest that hindgut- and frass-derived volatiles play important roles in bark beetle chemical communication. In the present study, we sought to identify volatile compounds shared between the hindgut and frass of *T. pilifer* that may contribute to adult behavioral responses. GC–MS analysis identified eight compounds common to both sources. Among these, the five monoterpenes α-pinene, 3-carene, D-limonene, camphene, and β-myrcene elicited significant electroantennographic responses and positive behavioral attraction, suggesting that these compounds may serve as key semiochemicals involved in the aggregation behavior of *T. pilifer*. Terpenes represent one of the most abundant classes of plant volatile organic compounds [33], and numerous herbivorous insects rely on these compounds for host location and behavioral regulation [34]. Consistent with previous studies, α-pinene and 3-carene, which elicited strong EAG responses in the present study, have been reported to attract a wide range of insect species [34,35,36]. These findings further support the ecological importance of terpene volatiles in mediating host recognition by bark beetles. Moreover, when present in hindgut- and frass-derived odor blends, these compounds may also contribute to intraspecific chemical communication and aggregation-related behaviors, as reported for other bark beetle species [22,37]. Interestingly, female adults consistently exhibited slightly greater EAG amplitudes than males, indicating a higher sensitivity to host-derived volatiles. This sexual difference may reflect the greater dependence of females on host chemical cues during host location, oviposition, and colonization. Consistent with this interpretation, previous studies have shown that host volatiles can enhance both electrophysiological and behavioral responses in females while simultaneously inducing the expression of multiple OBPs [15,38], suggesting that females may require a greater capacity for detecting and discriminating host-associated odor cues.

Notably, the EAG responses of most tested compounds exhibited a concentration-dependent pattern, although stronger responses were not necessarily associated with higher concentrations. For example, α-pinene and camphene elicited pronounced antennal responses at a relatively low concentration (0.001 μg/μL), whereas D-limonene remained highly active over a broader concentration range. These observations suggest that different volatile compounds may possess distinct optimal stimulus concentrations, while excessive concentrations may reduce olfactory sensitivity through receptor adaptation or sensory inhibition. Similar dose-dependent behavioral responses have been reported in other bark beetles, in which the effectiveness of semiochemical lures is influenced by multiple factors, including volatile release rate, ambient concentration, and lure composition, and optimal attraction is achieved only within an appropriate concentration range [39]. Collectively, these findings indicate that host-derived volatile compounds are likely to function effectively only within specific concentration windows under natural conditions.

Olfaction is a primary mechanism by which insects detect and discriminate environmental chemical signals, and this process largely depends on the interactions between odorant-binding proteins (OBPs) and odor molecules. OBPs are small soluble proteins that are highly enriched in insect antennae and function as important carriers for the recognition and transport of odorants within the peripheral olfactory system [10]. In the present study, candidate OBPs of *T. pilifer* were systematically characterized through expression profiling, phylogenetic analysis, and conserved motif analysis. Both transcriptome-based expression profiling and RT-qPCR revealed that TpilOBP5, TpilOBP16, and TpilOBP29 were highly expressed in the antennae of both female and male adults, with TpilOBP29 exhibiting the most pronounced antenna-enriched expression pattern. These expression profiles suggest that the three OBPs may be involved in the detection of host-derived volatiles or other semiochemical signals. Such antenna-biased expression is consistent with the typical characteristics of olfactory-related OBPs that participate in peripheral odor detection in insects [11]. Although the abdomen is a heterogeneous tissue, it was selected as the calibrator tissue because it represents a non-olfactory tissue and allows relative comparison of OBP expression among different tissues. The close phylogenetic relationships between TpilOBP5, TpilOBP16, and TpilOBP29 and the functionally characterized PparOBP1, TyunOBP3, and TyunOBP2, respectively, further support the potential roles of these candidate OBPs in host volatile recognition [40,41,42]. In addition, all three candidate OBPs belonged to the Minus-C OBP subfamily and formed well-supported clades with homologous OBPs from related scolytine species, indicating a relatively conserved evolutionary origin. Previous studies have shown that Minus-C OBPs in coleopteran insects are frequently associated with host volatile recognition and chemical communication and often exhibit relatively conserved functional and evolutionary characteristics [11,42,43,44]. Moreover, conserved motif analysis revealed that the three candidate OBPs shared several highly conserved motifs (Motifs 1–3), whereas differences were observed in the composition and arrangement of additional motifs (Figure 6). These results suggest that the candidate OBPs may maintain a conserved structural framework while modulating ligand-binding properties through local sequence variation, thereby facilitating the recognition of diverse chemical cues. Such structural differences may contribute to functional differentiation among TpilOBPs in odor recognition and ligand selectivity [10].

Fluorescence competitive binding assays demonstrated that all three candidate OBPs (TpilOBP5, TpilOBP16, and TpilOBP29) were capable of binding the five EAG-active terpene compounds, although their ligand-binding spectra differed considerably. Among them, TpilOBP29 exhibited the broadest ligand-binding spectrum and the highest binding affinities (Ki = 3.16–10.16 μM), suggesting that this protein plays a prominent role in the recognition of terpene volatiles and may contribute substantially to the perception of host-derived chemical cues. In contrast, TpilOBP16 and TpilOBP5 displayed comparatively weaker binding affinities and greater ligand selectivity, indicating functional differentiation among TpilOBPs during odor recognition. Similar terpene-binding properties have also been reported in other forest pests. For example, MaltOBPs from the pine sawyer beetle Monochamus alternatus have been shown to bind several pine-associated monoterpenes, including α-pinene, limonene, camphene, and β-myrcene. In addition, some OBPs from the European spruce bark beetle Ips typographus have been demonstrated to recognize host-derived volatiles such as α-pinene, β-pinene, and limonene. These studies suggest that OBP-mediated recognition of conifer-associated terpenes may represent a relatively conserved olfactory adaptation among wood-feeding Coleoptera. However, differences in ligand selectivity among OBPs further indicate that functional diversification of OBPs contributes to the discrimination of complex odor cues [45,46].

This functional divergence was further supported by fluorescence competitive binding assays using additional volatile compounds selected from the molecular docking analysis. TpilOBP29 maintained relatively high binding affinities toward several non-target volatiles, particularly α-caryophyllene (Ki = 1.94 μM), whereas TpilOBP16 exhibited moderate affinities toward compounds such as α-ionone (Ki = 6.15 μM) and (−)-verbenone (Ki = 10.31 μM). By comparison, TpilOBP5 generally showed weaker binding affinities toward the tested ligands. Collectively, these results indicate that TpilOBP29 functions as a broadly tuned OBP, whereas TpilOBP16 and TpilOBP5 exhibit relatively narrower ligand selectivity. Such functional specialization is consistent with the combinatorial coding model of insect olfaction, in which multiple OBPs with distinct but partially overlapping ligand specificities cooperate to encode complex odor information [47,48]. Although fusion tags were retained during the ligand-binding assays, MBP is primarily a maltose/maltodextrin-binding protein whose ligand recognition depends mainly on hydrogen-bonding and polar interactions with carbohydrate molecules [49]. Therefore, the hydrophobic monoterpenes tested in this study are unlikely to interact strongly with the MBP domain. Furthermore, the distinct ligand-binding profiles among TpilOBP5, TpilOBP16, and TpilOBP29, including their differential affinities toward individual terpenes, indicate that the observed fluorescence signals mainly reflect the intrinsic ligand recognition properties of TpilOBPs rather than nonspecific interactions from fusion tags. To further elucidate the molecular basis of ligand recognition, homology modeling and molecular docking analyses were performed to characterize the binding modes of the candidate OBPs. The docking results indicated that terpene ligands were primarily accommodated within hydrophobic binding cavities formed by conserved residues, including Phe, Val, Leu, and Ala, and were stabilized mainly through hydrophobic interactions, van der Waals forces, and π–alkyl interactions. This binding pattern is consistent with the canonical ligand recognition mechanism reported for insect OBPs that interact with hydrophobic terpene compounds [23,50]. Although all three candidate OBPs possessed the characteristic hydrophobic binding cavity typical of insect OBPs, differences in the integrity of the hydrophobic core and the composition of aromatic residues were associated with distinct ligand-binding properties. TpilOBP29 contained a more continuous hydrophobic core and a greater abundance of aromatic residues, which likely account for its stronger binding affinity and broader ligand spectrum. In contrast, TpilOBP16 exhibited intermediate binding capacity, whereas TpilOBP5 showed the weakest ligand-binding performance, possibly because its more discontinuous hydrophobic core and the presence of polar residues, such as Lys81 and Arg118, reduce the hydrophobic stability of the binding pocket. These findings suggest that subtle differences in the physicochemical microenvironment of the binding cavity may represent an important structural basis underlying ligand selectivity and functional divergence among TpilOBPs [31].

Notably, the molecular docking results were largely consistent with those obtained from the fluorescence competitive binding assays. The two approaches showed similar trends in ligand affinity rankings, supporting the reliability of molecular docking for predicting ligand-binding preferences and relative binding affinities of candidate OBPs. Nevertheless, discrepancies were observed for a few ligands. For example, although D-limonene exhibited a relatively modest docking score with TpilOBP5 (−5.0 kcal/mol), it displayed a comparatively strong binding affinity in the fluorescence assay (Ki = 2.91 μM). Such differences are likely attributable to the inherent limitations of molecular docking, which is based on static protein structures and therefore cannot fully capture protein conformational flexibility or the dynamic nature of protein–ligand interactions in solution [42,51]. Together, these findings suggest that TpilOBP5, TpilOBP16, and TpilOBP29 contribute differently to terpene perception in *T. pilifer*, providing new insights into the molecular basis of olfactory recognition in this species.

## 5. Conclusions

This study identified five behaviorally active monoterpenes shared between the hindgut and frass of *Tomicus pilifer* and demonstrated that they are likely involved in host location and chemical communication. Three candidate odorant-binding proteins, TpilOBP5, TpilOBP16, and TpilOBP29, were functionally characterized through expression profiling, fluorescence competitive binding assays, and molecular docking analyses. Among them, TpilOBP29 exhibited the broadest ligand-binding spectrum and the highest binding affinity toward terpene volatiles, whereas TpilOBP5 and TpilOBP16 showed relatively greater ligand selectivity, indicating functional differentiation among these OBPs. Structural analyses further suggested that differences in the hydrophobic binding cavity contribute to ligand-binding specificity. These findings provide new insights into the molecular basis of olfactory recognition in *T. pilifer* and offer candidate molecular targets for the development of olfaction-based, environmentally sustainable management strategies.

## Figures and Tables

**Table 1:** Common volatile compounds identified in hindgut and frass samples of male and female *Tomicus pilifer* adults.

Compound	ID	RT, min	Peak Area Percentage (%)			
			Female Hindgut	Male Hindgut	Female Frass	Male Frass
α-Pinene	MS	5.224	0.205 ± 0.018	0.173 ± 0.015	8.808 ± 0.624	4.847 ± 0.382
Camphene	MS	5.562	0.190 ± 0.016	0.039 ± 0.016	1.226 ± 0.091	2.515 ± 0.184
β-Myrcene	MS	6.54	0.356 ± 0.029	0.226 ± 0.017	2.128 ± 0.156	3.028 ± 0.243
3-Carene	MS	6.998	0.307 ± 0.022	0.052 ± 0.005	1.441 ± 0.113	2.341 ± 0.196
D-Limonene	MS	7.45	0.387 ± 0.031	0.174 ± 0.014	1.645 ± 0.137	10.601 ± 0.851
Pentacosane	MS	16.13	0.147 ± 0.012	0.545 ± 0.043	12.255 ± 0.856	11.013 ± 0.792
Hexadecane	MS	23.466	0.253 ± 0.021	0.462 ± 0.038	1.371 ± 0.102	5.036 ± 0.351
Octacosane	MS	24.862	0.363 ± 0.028	1.034 ± 0.087	1.209 ± 0.097	5.424 ± 0.412

**Table 2:** Predicted binding energies of different volatile ligands docked with TpilOBP5, TpilOBP16, and TpilOBP29.

Compound Name	CAS Number	Purity (%)	Resource	Binding Energy (kcal/mol)		
				TpilOBP5	TpilOBP16	TpilOBP29
3-Carene	13466-78-9	≥98%	Aladdin	−4.7	−6.2	−7.5
Camphene	79-92-5	≥98%	Aladdin	−4.7	−6	−7.6
β-myrcene	123-35-3	≥98%	Aladdin	−4.7	−5.8	−6.3
D-Limonene	5989-27-5	≥95%	Aladdin	−5	−6.2	−7.3
α-Pinene	7785-70-8	≥98%	Aladdin	−4.9	−6.1	−7.6
β-caryophyllene	87-44-5	≥98%	Yuanye	−6.4	−8.7	−9.9
(+)-beta-Pinene	19902-08-0	≥95%	Aladdin	−4.8	−5.9	−7.4
Isolongifolene	1135-66-6	≥95%	Yuanye	−5.8	−7.7	−9.7
(+)-a-Longipinene	5989-08-2	≥95%	Yuanye	−6.1	−7.9	−9.6
(+)-Sativene	3650-28-0	≥98%	Macklin	−6.3	−8	−9.4
β-Farnesene	18794-84-8	≥95%	Yuanye	−5.7	−7.1	−7.8
(−)-beta-Pinene	18172-67-3	≥98%	Aladdin	−4.8	−6.2	−7.5
(−)-a-phellandrene	4221-98-1	≥98%	Aladdin	−5	−6.4	−7.4
a-Phellandrene	99-83-2	≥98%	Yuanye	−5.1	−6.3	−7.5
a-Terpinolene	586-62-9	≥95%	Aladdin	−5.2	−6.8	−7.2
Frontalin	28401-39-0	≥95%	Aladdin	-	−5.4	−6.4
(a)-lonone	127-41-3	≥98%	Aladdin	−5.7	−7.4	−7.4
(4S)-cis-Verbenol	18881-04-4	≥98%	Aladdin	−4.7	−6.5	−6.5
(−)-Fenchone	4695-62-9	≥98%	Aladdin	−5.1	−6.1	-
(−)-Verbenone	1196-01-6	≥98%	Aladdin	−4.7	−6.4	-
(+)-Longifolene	475-20-7	≥95%	Aladdin	−6.1	−7.8	−9.9

**Table 3:** Fluorescence competitive binding parameters of recombinant TpilOBP5, TpilOBP16, and TpilOBP29 with terpene compounds.

Ligand	TpilOBP5		TpilOBP16		TpilOBP29	
	IC50	Ki	IC50	Ki	IC50	Ki
3-Carene	13.71 ± 0.82	12.86 ± 0.73	5.844 ± 0.31	5.13 ± 0.28	9.728 ± 0.57	8.94 ± 0.51
Camphene	15.34 ± 0.91	14.39 ± 0.82	1.092 ± 0.06	0.96 ± 0.05	4.731 ± 0.26	4.35 ± 0.24
β-myrcene	12.57 ± 0.75	11.79 ± 0.68	16.92 ± 1.03	14.84 ± 0.91	4.681 ± 0.24	4.30 ± 0.22
D-Limonene	3.103 ± 0.16	2.91 ± 0.14	3.668 ± 0.19	3.22 ± 0.17	11.05 ± 0.67	10.16 ± 0.62
α-Pinene	4.671 ± 0.25	4.38 ± 0.23	6.331 ± 0.35	5.55 ± 0.31	3.434 ± 0.19	3.16 ± 0.18
(+)-Longifolene	10.74 ± 0.63	10.08 ± 0.59	6.099 ± 0.32	5.35 ± 0.29	3.264 ± 0.17	3.00 ± 0.16
Isolongifolene	9.393 ± 0.52	8.81 ± 0.48	4.356 ± 0.23	3.82 ± 0.21	6.649 ± 0.38	6.11 ± 0.35
α-Ionone	9.667 ± 0.56	9.07 ± 0.53	7.011 ± 0.39	6.15 ± 0.36	7.174 ± 0.41	6.59 ± 0.38
β-caryophyllene	5.959 ± 0.33	5.59 ± 0.30	4.575 ± 0.24	4.01 ± 0.22	2.110 ± 0.11	1.94 ± 0.09
(E)-β-Farnesene	7.746 ± 0.45	7.26 ± 0.42	8.929 ± 0.50	7.83 ± 0.45	2.888 ± 0.15	2.65 ± 0.14
a-Phellandrene	9.134 ± 0.51	8.57 ± 0.49	9.222 ± 0.52	8.09 ± 0.47	8.015 ± 0.44	7.36 ± 0.41
(−)-Verbenone	59.84 ± 3.48	56.10 ± 3.25	8.416 ± 0.48	7.38 ± 0.44	11.23 ± 0.71	10.31 ± 0.68

Note: Data are the mean ± S.E.

## Data Availability

The nucleotide sequences of TpilOBP5, TpilOBP16, and TpilOBP29 have been deposited in the NCBI GenBank database under accession numbers PZ717158, PZ717159, and PZ717160, respectively. Other datasets and raw data analyzed in this study are available within the article and Supplementary Materials, or can be obtained from the corresponding author upon reasonable request.

## Supplementary Materials

The following supporting information can be downloaded at: https://www.mdpi.com/article/10.3390/insects17080810/s1, Figure S1: Structure evaluation of Ramachandran. Figure S2: Model quality estimations of OBPs. Figure S3: Molecular docking visualization of three candidate OBPs. Figure S4: Molecular docking visualization of three candidate OBPs. Table S1: The detailed sequence data and the best blastx match. Table S2: Information about the organic compounds. Table S3: Primers used for RT-PCR. Table S4: Primers used for RT-qPCR. Table S5: The ligand-binding ability of three OBPs. Table S6: Molecular docking binding site analysis. Table S7: Primers used for RT-PCR. Table S8: Primers used for RT-qPCR. Table S9: The binding ability of three OBPs to ligands. Table S10: Molecular docking binding site analysis.
