# Supplementary material for: Molecular Basis of Behaviorally Active Terpenoid Volatile Recognition by Odorant-Binding Proteins in Tomicus pilifer
Source: Insects. 2026 Aug 4;17(8):810. doi: 10.3390/insects17080810 (PMC13513343; doi:10.3390/insects17080810)
Supplement: Supplementary file 1 [file insects-17-00810-s001.zip › insects-4448048-supplementary.pdf]

Supplementary materials for

Functional characterization of three odorant-binding proteins involved in the recognition of hindgut- and feces-derived terpenoid volatiles in the bark beetle *Tomicus pilifer*

List of contents:

Figure S1. Structure evaluation of Ramachandran.

Figure S2. Model quality estimations of OBPs.

Figure S3. Molecular docking visualization of three candidate OBPs

Figure S4. Molecular docking visualization of three candidate OBPs

Table S1. The detailed sequence data and the best blastx match

Table S2. Information about the organic compounds

Table S3. Primers used for RT-PCR

Table S4. Primers used for RT-qPCR

Table S5. The ligand-binding ability of three OBPs

Table S6. Molecular docking binding site analysis

Table S7. Primers used for RT-PCR

Table S8. Primers used for RT-qPCR

Table S9. The binding ability of three OBPs to ligands

Table S10. Molecular docking binding site analysis

Figure S1. Structure evaluation of Ramachandran.

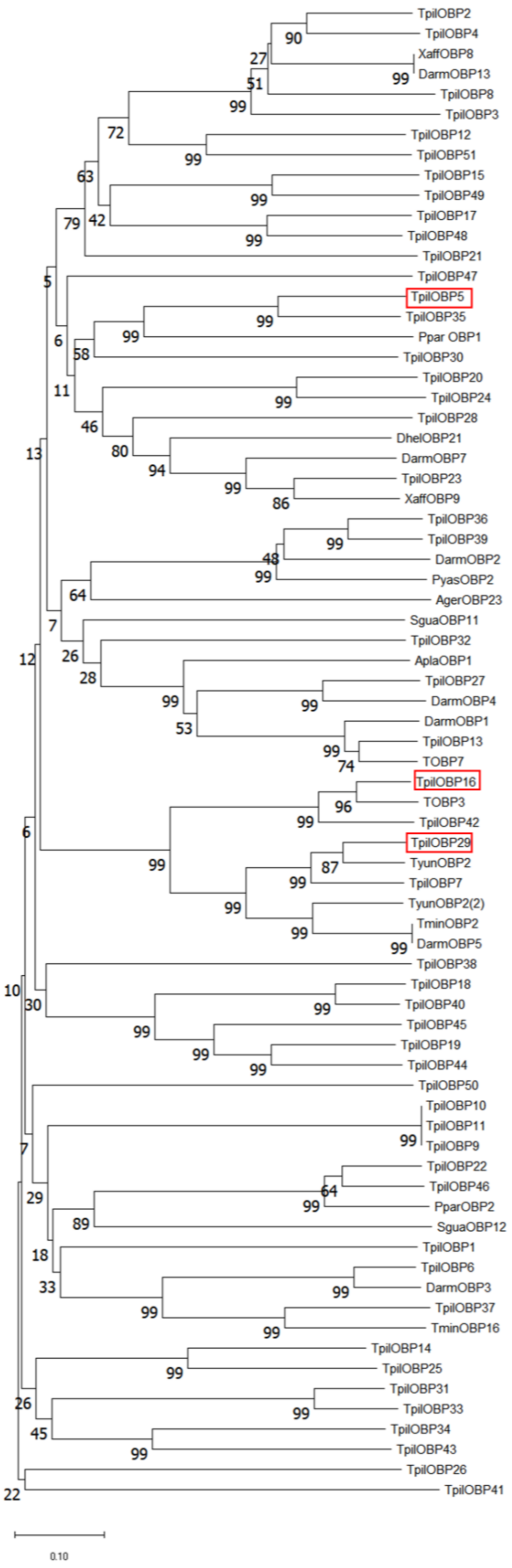

Figure S2. Model quality estimations of OBPs.

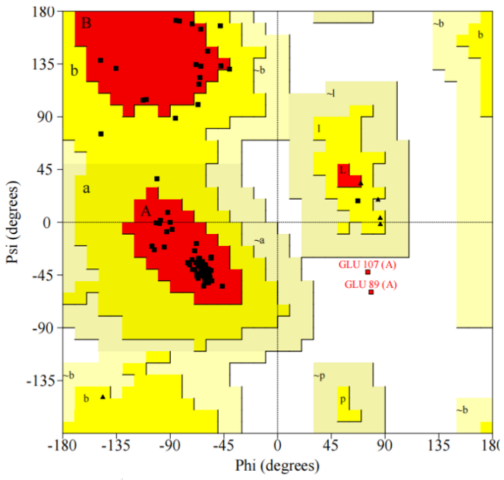

TpilOBP5

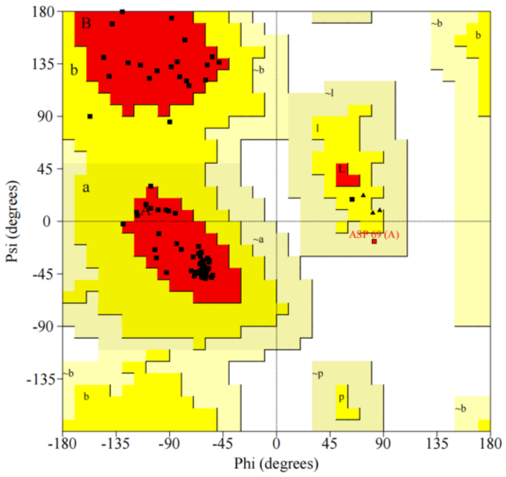

TpilOBP16

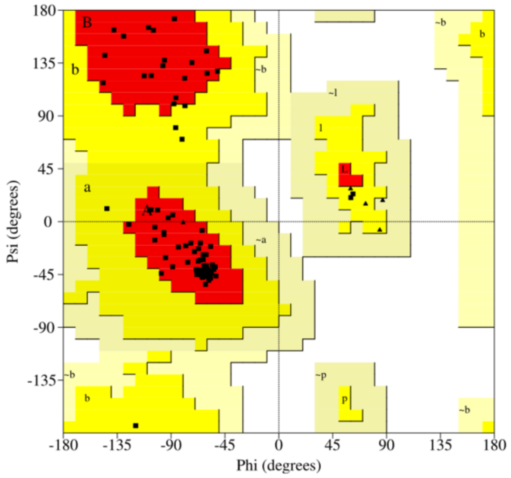

TpilOBP29

Figure S3. Molecular docking visualization of three candidate OBPs

TpilOBP5

Models

The following model was built (see Materials and Methods "Model Building"):

Model #01	File	Built with	Oligo-State	Ligands	GMQE
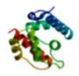	PDB	ProMod3 3.6.0	monomer	None	0.93

Template	Seq Identity	Oligo-state	QSQE	Found by	Method	Resolution	Seq Similarity	Range	Coverage	Description
A0A4P2HSL7.1.A	91.06	monomer	-	AFDB search	AlphaFold v2	-	0.60	1 - 123	1.00	Odorant binding protein 1

TpilOBP16

Models

The following model was built (see Materials and Methods "Model Building"):

Model #01	File	Built with	Oligo-State	Ligands	GMQE
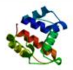	PDB	ProMod3 3.6.0	monomer	None	0.90

Template	Seq Identity	Oligo-state	QSQE	Found by	Method	Resolution	Seq Similarity	Range	Coverage	Description
A0A4P2HQ85.1.A	91.89	monomer	-	AFDB search	AlphaFold v2	-	0.59	1 - 111	1.00	Odorant binding protein 3

TpilOBP29

Models

The following model was built (see Materials and Methods "Model Building"):

Model #01	File	Built with	Oligo-State	Ligands	GMQE
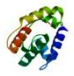	PDB	ProMod3 3.6.0	monomer	None	0.92

Template	Seq Identity	Oligo-state	QSQE	Found by	Method	Resolution	Seq Similarity	Range	Coverage	Description
A0A4P2HIY5.1.A	91.96	monomer	-	AFDB search	AlphaFold v2	-	0.61	1 - 112	1.00	Odorant binding protein 2

Figure S4. Molecular docking visualization of three candidate OBPs

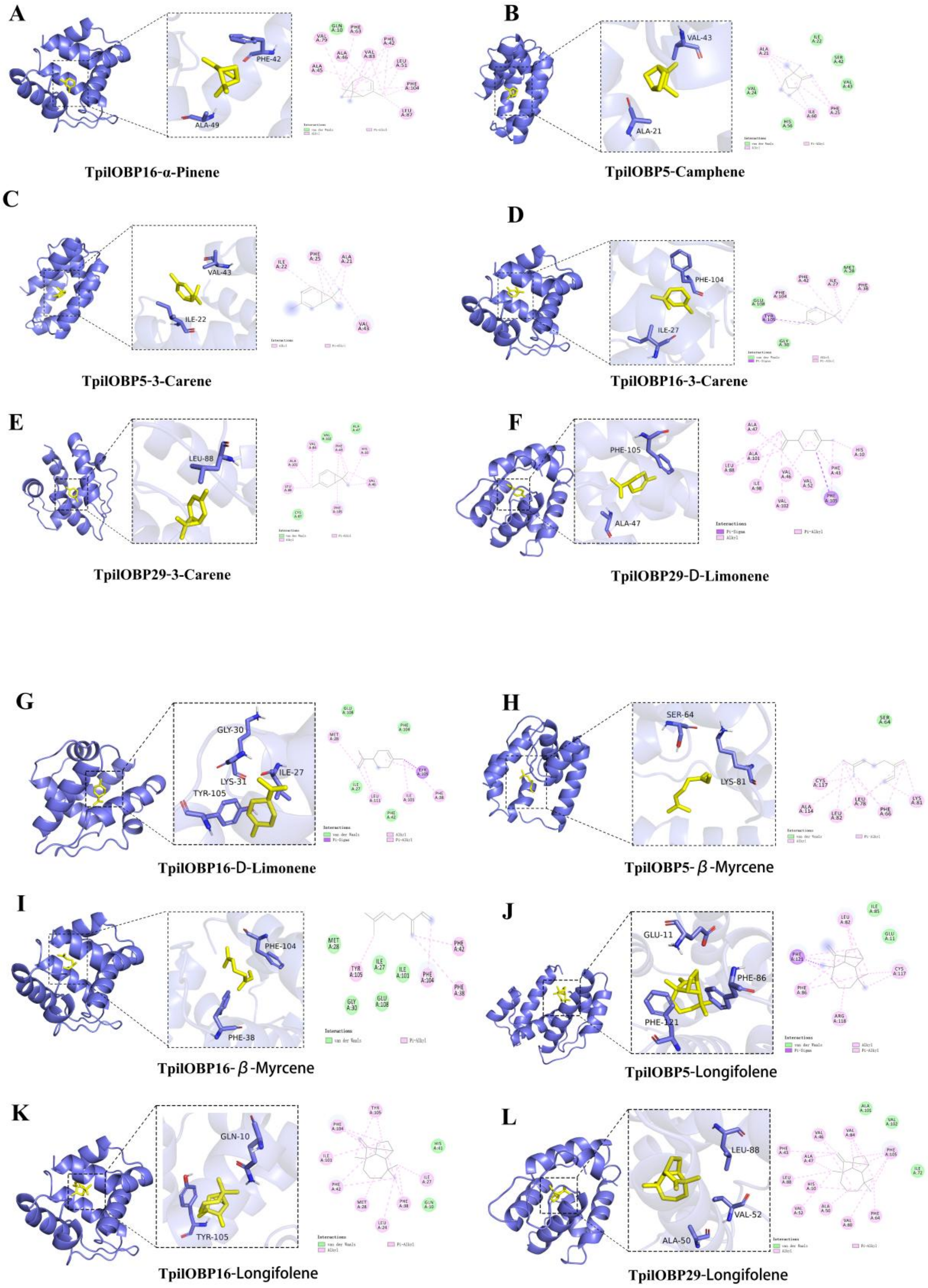
A
PHE-42
ALA-49
TpilOBP16-α-Pinene
B
VAL-43
ALA-21
TpilOBP5-Camphene
C
VAL-43
ILE-22
TpilOBP5-3-Carene
D
PHE-104
ILE-27
TpilOBP16-3-Carene
E
LEU-88
TpilOBP29-3-Carene
F
PHE-105
ALA-47
TpilOBP29-D-Limonene
G
GLY-30
LYS-31
ILE-27
TYR-105
TpilOBP16-D-Limonene
H
SER-64
LYS-81
TpilOBP5-β-Myrcene
I
PHE-104
PHE-38
TpilOBP16-β-Myrcene
J
GLU-11
PHE-86
PHE-121
TpilOBP5-Longifolene
K
GLN-10
TYR-105
TpilOBP16-Longifolene
L
LEU-88
VAL-52
ALA-50
TpilOBP29-Longifolene

L

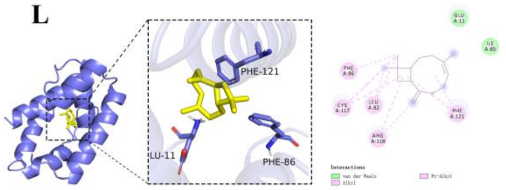

OBP5-E-Caryophyllene

M

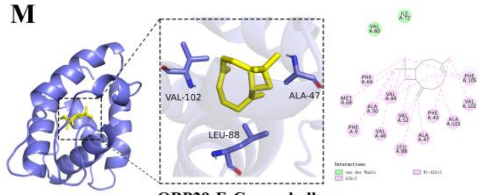

OBP29-E-Caryophyllene

Table S1. The detailed sequence data and the best blastx match

Compound	RT,min	Area(Ab*s)	Relative content(%)	Similarity	CAS number
Arsenous acid, tris(trimethylsilyl) ester	30.372	1255965	0.423088	47	55429-29-3
Ginsenol	29.016	1253582	0.422286	43	117591-80-7
Anthracene, 9,10-dihydro-9,9,10-trimethyl-	29.668	1210960	0.407928	49	109236-33-7
Ethyl 2-(2-chloroacetamido)-3,3,3-trifluoro-2-(3-fluoroanilino)propionate	29.274	1159101	0.390458	43	318298-84-9
1H-imidazole-2-methanol, 1-decyl-	29.148	1158735	0.390335	38	124777-84-0
D-Limonene	7.467	1150047	0.387408	92	5989-27-5
Bicyclo[2.2.1]heptane-2-carboxylic acid, methyl ester	27.62	1133313	0.381771	43	35520-81-1
[1,2,4]Triazolo[1,5-a]pyrimidine-6-carboxylic acid, 4,7-dihydro-7-imino-, ethyl ester	28.553	1115138	0.375649	25	92673-40-0
Pregn-5-ene-3,20-diol, (3.alpha.)-, 2TMS derivative	28.61	1104358	0.372017	27	55515-25-8
2-Methylhexacosane	24.599	1078575	0.363332	64	1561-02-0
Octacosane	24.931	1076376	0.362591	43	630-02-4
Carbonic acid, eicosyl vinyl ester	25.039	1061149	0.357462	41	2243791-78-6
.beta.-Myrcene	6.77	1060149	0.356462	45	123-35-3
2-(Acetoxymethyl)-3-(methoxycarbonyl)biphenylene	29.92	1055486	0.355554	47	93103-70-9
1,2,5-Oxadiazol-3-amine, 4-(4-methoxyphenoxy)-	29.771	1018111	0.342964	43	292856-68-9
Dodecane, 1,12-dibromo-	24.959	1015787	0.342181	49	3344-70-5
1,2-Benzenediol, 3,5-bis(1,1-dimethylethyl)-	30.103	1013408	0.34138	50	1020-31-1
Cholest-23-ene, (5.beta.)-	27.797	936221	0.315378	50	30658-62-9
Silicic acid, diethyl bis(trimethylsilyl) ester	30.996	924896	0.311563	78	3555-45-1

3-Carene	7.69	908285	0.3072057	44	13466-78-9
2-Tridecylfuran	24.788	901220	0.303588	43	25346-22-9
Aspidospermidin-17-ol, 1-acetyl-19,21-epoxy-15,16-dimethoxy-	24.759	893944	0.301137	46	2122-26-1
Benzo[h]quinoline, 2,4-dimethyl-	28.924	870498	0.293239	30	605-67-4
Tetradecane	18.699	794148	0.267519	93	629-59-4
Hexadecane	23.466	752218	0.253394	94	544-76-3
2,3,4-Trimethoxyphenylacetonitrile	29.079	748957	0.252296	30	68913-85-9
Eicosane	24.215	695153	0.234171	46	112-95-8
4-Hydroxy-.beta.-ionone	29.113	644279	0.217034	35	543-85-9
Avocadenofuran	24.822	643097	0.216636	44	25346-24-1
Cyclopentasiloxane, decamethyl-	10.986	636928	0.214558	78	541-02-6
Sulfurous acid, butyl heptadecyl ester	22.842	633089	0.213264	83	1000309-18-4
2-Methylhexacosane	24.33	622341	0.209644	58	1561-02-0
.alpha.-Pinene	5.224	608178	0.204873	94	80-56-8
1,4-Benzenediol, 2,5-bis(1,1-dimethylethyl)-	29.222	592467	0.19958	43	88-58-4
Camphene	5.545	562710	0.189556	94	79-92-5
Cyclohexasiloxane, dodecamethyl-	16.382	545250	0.183675	87	540-97-6
1,2,5-Oxadiazol-3-amine, 4-(4-methoxyphenoxy)-	29.645	541589	0.182441	47	292856-68-9
Tridecane, 1-iodo-	14.602	540522	0.182082	80	35599-77-0
4-(3-Nitrophenyl)-1,2,3-thiadiazole	12.331	537060	0.180916	33	77414-51-8
2-(Acetoxymethyl)-3-(methoxycarbonyl)biphenylene	31.065	444802	0.149837	47	93103-70-9
Dotriacontane, 1-iodo-	23.849	441699	0.148792	72	62154-83-0
Pentacosane	16.13	436332	0.146984	72	629-99-2
Cyclohexanecarboxamide, N-furfuryl-	28.696	432339	0.145639	30	6341-32-8
1-Octadecanesulphonyl chloride	21.955	428051	0.144195	90	10147-41-8

Table S2. Information about the organic compounds

Compound	RT, min	Area (Ab*s)	Relative content(%)	Similarity	CAS number
Avocadynofuran	26.035	6023932	1.040518	59	24708-33-6
Octacosane	24.862	5988604	1.034415	83	630-02-4
3-Eicosyne	25.835	5914274	1.021576	38	61886-66-6
1-Bromo-11-iodoundecane	29.01	3307965	0.571387	55	139123-69-6
Pentacosane	22.688	3157001	0.545311	80	629-99-2
Methoxyacetic acid, 2-pentadecyl ester	24.793	2890437	0.499267	38	959264-10-9
Tetradecane	18.699	2705015	0.467239	97	629-59-4
Hexadecane	23.466	2677049	0.462408	95	544-76-3
Liguloxide	29.073	1505790	0.260096	47	21764-22-7
Triacontyl acetate	28.261	1502014	0.259444	35	41755-58-2
Octatetracontane, 1-iodo-	24.439	1488947	0.257187	60	40710-70-1
Tetradecane, 1-iodo-	14.597	1488364	0.257086	90	19218-94-1
.beta.-Myrcene	6.53	1308049	0.225940	45	123-35-3
Tetracosane	24.221	1120893	0.193613	53	646-31-1
Pentadecane	21.961	1119428	0.19336	95	629-62-9
Eicosane	22.064	1068034	0.184482	90	112-95-8
Hexacosane	21.738	1017670	0.175783	90	630-01-3
D-Limonene	7.45	1005094	0.173611	95	

						5989-27-5
.alpha.-Pinene	5.224	999953	0.172723	91	80-56-8	
3-Carene	6.992	301374	0.052057	55	13466-78-9	
10-Methylnonadecane	21	275652	0.047614	50	56862-62-5	
Undecane	8.377	272775	0.047117	78	1120-21-4	
Tridecane	21.125	265707	0.045896	59	629-50-5	
Undecane, 4,6-dimethyl-	16.697	261307	0.045136	64		17312-82-2
Heptadecane, 4-methyl-	20.788	257927	0.044552	59	26429-11-8	
Tetracontane, 3,5,24-trimethyl-	19.129	246774	0.042625	59		55162-61-3
Pentatriacontane, 1-bromo-	23.592	242607	0.041906	53		62108-51-4
6-(Methylthio)hexanenitrile	12.331	237669	0.041053	10	72931-29-4	
Camphene	5.562	224002	0.038692	81	79-92-5	
.beta.-Pinene	6.191	223714	0.038642	53		127-91-3
2-Methyltetracosane	17.418	62751	0.010839	59		1560-78-7
Avocadynofuran	26.035	6023932	1.040518	59	24708-33-6	
cis-1-Chloro-9-octadecene	27.397	6001643	1.036668	89	16507-61-2	
Octacosane	24.862	5988604	1.034415	83		630-02-4
3-Eicosyne	25.835	5914274	1.021576	38	61886-66-6	
1-Bromo-11-iodoundecane	29.01	3307965	0.571387	55	139123-69-6	
1,4-Benzenediol, 2,5-bis(1,1-dimethylethyl)-	29.331	3237249	0.559172	45	88-58-4	
1,2-Bis(trimethylsilyl)be	30.1	32284	0.55	41	17151-09-6	

nzene	6	18	7647			
Pentacosane	22.688	3157001	0.545311	80	629-99-2	
Methoxyacetic acid, 2-pentadecyl ester	24.793	2890437	0.499267	38	959264-10-9	
2'-Hydroxy-5'-methylacetophenone, TMS derivative	30.286	2819258	0.486972	38	1450-72-2	
1,2-Benzenediol, 3,5-bis(1,1-dimethylethyl)-	30.515	2812462	0.485798	46	1020-31-1	
Xanthen-9-one, 1-hydroxy-3,5,8-trimethoxy-	28.455	2809360	0.485263	49	49599-09-9	
Tetradecane	18.699	2705015	0.467239	97	629-59-4	
Cyclotrisiloxane, hexamethyl-	29.806	2691606	0.464923	38	541-05-9	
Hexadecane	23.466	2677049	0.462408	95	544-76-3	
Liguloxide	29.073	1505790	0.260096	47	21764-22-7	
Triacontyl acetate	28.261	1502014	0.259444	35	41755-58-2	
Octatetracontane, 1-iodo-	24.439	1488947	0.257187	60	40710-70-1	
Tetradecane, 1-iodo-	14.597	1488364	0.257086	90	19218-94-1	
.beta.-Myrcene	6.53	1308049	0.225940	45		123-35-3
Tetracosane	24.221	1120893	0.193613	53	646-31-1	
Pentadecane	21.961	1119428	0.19336	95		629-62-9
Tetradecane	16.13	1098250	0.189701	78	629-59-4	

Table S3. Primers used for RT-PCR

Compound	RT,min	Area (Ab*s)	Relative content(%)	Similarity	CAS Number
Bornyl acetate	14.763	83772549	19.833	99	000076-49-3
Longifolene	18.745	48941682	11.5869	99	000475-20-7
Caryophyllene	19.277	43518160	10.3029	99	000087-44-5
.alpha.-Pinene	5.218	37205621	8.8084	96	000080-56-8
Tricyclo[5.4.0.0(2,8)]undec-9-ene, 2,6,6,9-tetramethyl-, (1R,2S,7R,8R)-	16.868	19249084	4.5572	99	005989-08-2
Camphene	5.539	13626786	1.2261	97	000079-92-5
Humulene	20.473	11997279	2.8403	97	006753-98-6
Bicyclo[3.1.1]heptane, 6,6-dimethyl-2-methylene-, (1S)-	6.174	10524332	2.4916	97	018172-67-3
endo-Borneol	11.106	9475269	2.2433	97	000507-70-0
.beta.-Myrcene	8.33	8988616	2.1279	98	000123-35-3
1,3,6,10-Cyclotetradecatetraene, 3,7,11-trimethyl-14-(1-methylethyl)-, [S-(E,Z,E,E)]-	26.767	7833695	1.8546	99	001898-13-1
D-Limonene	7.461	6951687	1.6458	98	005989-27-5
endo-2-Methylbicyclo[3.3.1]nonane	23.58	6826465	1.6162	83	042558-37-2
Octadecane	28.621	6366442	1.5072	91	000593-45-3
3-Carene	6.439	24291071	1.4405	96	013466-78-9

Caryophyllene oxide	23.254	5721943	1.3547	89	001139-30-6
1,19-Eicosadiene	31.002	5496677	1.3013	86	014811-95-1
.gamma.-Muurolene	22.201	5395206	1.2773	90	030021-74-0
.gamma.-Terpinene	6.992	4575568	1.0833	95	000099-85-4
Naphthalene, 1,2,3,5,6,8a-hexahydro-4,7-dimethyl-1-(1-methylethyl)-, (1S-cis)-	22.379	3718415	0.8803	98	000483-76-1
Methyl abietate	28.558	3597963	0.8518	55	000127-25-3
1-Naphthalenecarboxylic acid, decahydro-1,4a-dimethyl-6-methylene-5-(3-methyl-2,4-pentadienyl)-, methyl ester, [1S-(1.alpha.,4a.alpha.,5.alpha.,8a.beta.)]-	28.604	3222328	0.7629	64	001235-39-8
4-Dehydroxy-N-(4,5-methylenedioxy-2-nitrobenzylidene)tyramine	29.794	2998014	0.7098	52	1000111-66-9
(1R,4aR,4bR,10aR)-7-Isopropyl-1,4a-dimethyl-1,2,3,4,4a,4b,5,6,10,10a-decahydrophenanthrene-1-carbaldehyde	23.157	2740801	0.6489	95	039029-41-9
Phenol, 2,2'-methylenebis[6-(1,1-dimethylethyl)-4-methyl-	28.02	2648769	0.6271	97	006704-50-3
(4E,8E,13E)-1-(2-Hydroxyethyl)-1,5,9-trimethylcyclotetradecatriene	28.387	2464162	0.5834	97	000119-47-1
.alpha.-Muurolene	27.648	2428666	0.575	84	1000426-96-0
1,3,6,10-Cyclotetradecatetraene, 3,7,11-trimethyl-14-(1-methylethyl)-, [S-(E,Z,E,E)]-	26.47	2315373	0.5482	64	000515-03-7
Thunbergol	26.104	2115326	0.5008	99	001898-13-1
(S,E)-8,12,15,15-Tetramethyl-4-methylenebicyclo[9.3.1]pentadeca-7,11-diene	27.683	1924511	0.4556	98	013508-03-7
Silane, dimethyl(4-methoxyphenoxy)isobutoxy-	21.921	1875871	0.4441	98	010208-80-7
Germacrene D	23.42	1811996	0.429	95	000465-24-7
Hexadecane	26.184	1802955	1.3714	96	001898-13-1
.alpha.-Cubebene	27.196	1797846	0.4256	62	025269-17-4
Benzo(e)pyrene, 9,10-dihydro-	27.082	1699252	0.4023	53	386223-19-4
cis-p-Mentha-2,8-dien-1-ol	29.388	16861	0.399	5	000465-

		51	2	5	24-7
Cyclohexane, 1-ethenyl-1-methyl-2-(1-methylethenyl)-4-(1-methylethylidene)-	26.887	1637687	0.3877	53	1000347-14-1
Phenol, 2-methyl-4-(1,1,3,3-tetramethylbutyl)-	21.417	1622634	0.3842	97	023986-74-5
Pentacosane	23.46	1568780	12.2554	94	000544-76-3
Tetracosane	17.772	1487835	0.3522	97	017699-14-8
Triacontane, 1-iodo-	27.488	1478959	0.3501	64	066788-01-0
Octacosane	27.242	1425774	1.2087	50	003886-78-0
.gamma.-Muurolene	17.532	1415169	0.335	97	001137-12-8
.alpha.-Farnesene	21.812	1335754	0.3162	91	003242-08-8
10s,11s-Himachala-3(12),4-diene	18.242	1271975	0.3011	97	003650-28-0
Octadecane	26.207	1173072	0.2777	43	002219-84-3
D-Glucose, diethyl mercaptal	21.869	1078687	0.2554	86	000629-99-2
Zonarene	27.826	1048374	0.2482	97	013601-88-2
Octadecane, 3-ethyl-5-(2-ethylbutyl)-	23.975	962943	0.228	86	000483-76-1
Hexadecane, 2-methyl-	28.221	935001	0.2214	97	000646-31-1
Ethanone, 2-(2-benzothiazolylthio)-1-(3,5-dimethylpyrazolyl)-	22.693	909896	0.2154	80	1000406-32-3
1-Hexacosene	24.53	881484	0.2087	91	000630-02-4
Germacrene D	23.798	873493	0.2068	70	000473-13-2
.delta.8(14)-Isopimaric acid	21.314	866406	0.2051	98	030021-74-0

Table S4. Primers used for RT-qPCR

Compound	RT,min	Area(Ab*s)	Relative content(%)	Similarity	CAS Number
Thunbergol	26.79	683950533	9.5875	91	025269-17-4
Abietic acid	28.787	545458299	7.6461	95	000514-10-3
Isopimaric acid	28.433	447163601	6.2682	98	005835-26-7
Longifolene	18.751	112565049	1.5779	99	000475-20-7
Octadecane	29.016	107956144	1.5133	80	000593-45-3
Heneicosane	29.388	97992159	1.3736	95	000629-94-7
Ferruginol	27.689	84902899	1.1901	91	000514-62-5
Caryophyllene	19.277	81013256	1.1356	99	000087-44-5
Octadecane, 1-chloro-	28.227	70255391	0.9848	98	003386-33-2
.delta.8(14)-Isopimaric acid	29.806	66325504	0.9297	92	000471-74-9
alpha.-Pinene	6.18	60410729	4.8468	94	000080-56-8
Caryophyllene oxide	23.254	54385978	0.7624	97	001139-30-6
D-Limonene	7.462	42932386	10.6018	99	005989-27-5
Octadecane, 1-chloro-	27.797	38511892	0.5399	97	003386-33-2
Heneicosane	30.161	36942685	0.5179	95	000629-94-7
Camphene	5.539	36737224	2.515	97	000079-92-5
Octacosane	29.76	30229106	5.4237	99	000630-02-4
Methyl dehydroabietate	28.118	30197590	0.4233	9	001235-74-1

				8	
3-Carene	6.998	24291071	2.3405	96	013466-78-9
Hexadecane	23.46	2597219	5.03644	94	000544-76-3
Pentacosane	14.608	895497	11.0126	86	000629-99-2
(-)-Carvone	13.412	787927	0.011	74	006485-40-1
Pinocarvone	11.026	745586	0.0105	87	030460-92-5
cis-Dihydrocarvone	12.268	734070	0.0103	45	003792-53-8
Thunbergol	28.787	545458299	7.6461	95	000514-10-3
Abietic acid	28.433	447163601	6.2682	98	005835-26-7
Isopimaric acid	28.753	293062329	4.1081	60	001235-39-8
Longifolene	29.016	107956144	1.5133	80	000593-45-3
Octadecane	26.184	104202040	1.4607	99	001898-13-1
Heneicosane	29.085	96740366	1.3561	64	082085-87-8
Ferruginol	26.109	84787638	1.1885	99	001898-13-1
Caryophyllene	28.227	70255391	0.9848	98	003386-33-2
Octadecane, 1-chloro-	29.806	66325504	0.9297	92	000471-74-9
.delta.8(14)-Isopimaric acid	27.832	65494890	0.9181	99	013601-88-2
alpha.-Pinene	23.254	54385978	0.7624	97	001139-30-6
Caryophyllene oxide	28.312	49628996	0.6957	70	003310-97-2
4-Isopropenyl-4,7-dimethyl-1-oxaspiro[2.5]octane	7.462	42932386	0.6018	99	005989-27-5
D-Limonene	29.342	41236814	0.578	42	010267-14-8
Octadecane, 1-chloro-	30.161	36942685	0.5179	95	000629-94-7
Heneicosane	5.539	36737224	0.515	9	000079-92-5

Camphene	27.391	35820890	0.5021	747	003564-54-3
17-Norkaur-15-ene, 13-methyl-, (8.beta.,13.beta.)-	29.308	35578941	0.4987	64	015798-13-7
Cyclooctane, 1,5-dimethyl-2,6-bis(methylene)-	29.76	30229106	0.4237	99	000630-02-4
Methyl dehydroabietate	30.052	28062651	0.3934	45	1000316-82-8
[1,4]Diazepane, 1,4-bis-(2-methylpyridin-4-yl)-	30.275	24587592	0.3447	80	001235-39-8
3-Carene	25.949	2757633	0.0387	90	070901-63-2
Hexadecane	9.047	2538065	0.0356	78	000586-62-9
Cyclohexene, 1-methyl-4-(1-methylethylidene)-	9.504	2446225	0.0343	60	018368-95-1
1,3,8-p-Menthatriene	19.003	2435763	0.0341	95	055732-78-0
(1R,5R)-1-Isopropyl-8-methyl-4-methylenespiro[4.5]dec-7-ene	8.886	925813	0.013	38	001005-64-7
(E)-1-Phenyl-1-butene	14.608	895497	0.0126	86	000629-99-2
Pentacosane	23.351	873596	0.0122	66	063181-42-0
2-Heptenoic acid, 7-(methylenecyclopropyl)-, methyl ester	13.412	787927	0.011	74	006485-40-1
(-)-Carvone	24.936	757782	0.0106	62	022599-96-8
Pinocarvone	13.882	745123	0.0104	86	053282-47-6
cis-Dihydrocarvone	28.593	1368033226	19.1768	93	010267-14-8
.beta.-Myrcene	8.33	8988616	3.0279	98	000123-35-3

Table S5. The ligand-binding ability of three OBPs

unigenes	Name	ORF（nt/aa）	species	gene description	E value	Identity (%)	Acc.number
TRINITY_DN19160_c0_g1	*TpilOBP5*	426|141	Tomicus yunnanensis	odorant binding protein 1	2.00E-88	91.49%	AMP19483.1
TRINITY_DN21727_c0_g1	*TpilOBP16*	456|135	Tomicus yunnanensis	odorant binding protein 3	1.00E-82	89.63%	AMP19485.1
TRINITY_DN4484_c0_g1	*TpilOBP29*	399|132	Tomicus yunnanensis	odorant binding protein 2	5.00E-75	90.15%	AMP19484.1

Table S6. Molecular docking binding site analysis

Compound name	CAS number	Purity (%)	Resource	Binding energy(kcal/mol)		
				TpilOBP5	TpilOBP16	TpilOBP29
3-Carene	13466-78-9	≥98%	Aladdin, Shanghai, China	-4.7	-6.2	-7.5
Camphene	79-92-5	≥98%	Aladdin, Shanghai, China	-4.7	-6	-7.6
β -Myrcene	123-35-3	≥98%	Aladdin, Shanghai, China	-4.7	-5.8	-6.3
D-Limonene	5989-27-5	≥95%	Aladdin, Shanghai, China	-5	-6.2	-7.3
α -Pinene	80-56-8	≥98%	Aladdin, Shanghai, China	-4.9	-6.1	-7.6
β -Caryophyllene	87-44-5	≥98%	Yuanye, Shanghai, China	-6.4	-8.7	-9.9
(+)- β -Pinene	19902-08-0	≥95%	Aladdin, Shanghai, China	-4.8	-5.9	-7.4
Isolongifolene	1135-66-6	≥95%	Yuanye, Shanghai, China	-5.8	-7.7	-9.7
(+)- α -Longipinene	5989-08-2	≥95%	Yuanye, Shanghai, China	-6.1	-7.9	-9.6
(+)-Sativene	3650-28-0	≥98%	Macklin, Shanghai, China	-6.3	-8	-9.4
β -Farnesene	18794-84-8	≥95%	Yuanye, Shanghai, China	-5.7	-7.1	-7.8
(-)- β -Pinene	18172-67-3	≥98%	Aladdin, Shanghai, China	-4.8	-6.2	-7.5
(-)- α -Phellandrene	4221-98-1	≥98%	Aladdin, Shanghai, China	-5	-6.4	-7.4

α-Phellandrene	99-83-2	≥98%	Yuanye, Shanghai, China	-5.1	-6.3	-7.5
Terpinolene	586-62-9	≥95%	Aladdin, Shanghai, China	-5.2	-6.8	-7.2
Frontalin	28401-39-0	≥95%	Aladdin, Shanghai, China	-	-5.4	-6.4
α-Ionone	127-41-3	≥98%	Aladdin, Shanghai, China	-5.7	-7.4	-7.4
(4S)-cis-Verbenol	18881-04-4	≥98%	Aladdin, Shanghai, China	-4.7	-6.5	-6.5
(-)-Fenchone	4695-62-9	≥98%	Aladdin, Shanghai, China	-5.1	-6.1	-
(-)-Verbenone	1196-01-6	≥98%	Aladdin, Shanghai, China	-4.7	-6.4	-
(+)-Longifolene	475-20-7	≥95%	Aladdin, Shanghai, China	-6.1	-7.8	-9.9

Table S7. Primers used for RT-PCR

Gene	Forward Primer（5’-3’）	Reverse Primer（5’-3’）
TpilO	CGAATTCGAGCTCCGTCGACGATTTT	TCGAGTGCGGCCGCAAGCTTCTAGT
BP5	GATATCAGCAAATTTAAAGAAT	TGTGGAATTCTTGACGACAG
TpilO	TGTACTTCCAATCCAATGCAGTATTTG	ATCCACTTCCAATGTTATTATTACAA
BP16	TGGCAGTCTTTTTGGTAT	CTTGTGCTCCGCAGAATAA
TpilO	TGTACTTCCAATCCAATGCAGATCAA	ATCCACTTCCAATGTTATTACTACAG
BP29	CGTCAAAAAGTGGTTGATT	TTTATGATCTTTTCCCAAA

Table S8. Primers used for RT-qPCR

Gene	Forward Primer（5’-3’）	Reverse Primer（5’-3’）
TpilOBP5	TGCTGTTGGCTGCTGTAGTG	GTGGCTCCTGTTTCTGCCAT
TpilOBP16	GCCCTGGTTAGAAAGTGCCT	CAACTTGTGCTCCGCAGAAT
TpilOBP29	GGCTCTGGCATTTGTTGTGA	TCCAAACAGGGTCGATGGAA
RPL18	CAGGCACATGAAAAAGCCCG	GTGAGCTTGGGTACTCCGTT

Table S9. The binding ability of three OBPs to ligands

Ligand	CAS number	TpiiOBP5（15.14）		TpiiOBP16(7.113)		TpiiOBP29(11.41)	
		IC50	Ki	IC50	Ki	IC50	Ki
3-Carene	13466-78-9	13.71 ± 0.82	12.86 ± 0.73	5.844 ± 0.31	5.13 ± 0.28	9.728 ± 0.57	8.94 ± 0.51
Camphene	79-92-5	15.34 ± 0.91	14.39 ± 0.82	1.092 ± 0.06	0.96 ± 0.05	4.731 ± 0.26	4.35 ± 0.24
Β-Myrcene	123-35-3	12.57 ± 0.75	11.79 ± 0.68	16.92 ± 1.03	14.84 ± 0.91	4.681 ± 0.24	4.30 ± 0.22
D-Limonene	5989-27-5	3.103 ± 0.16	2.91 ± 0.14	3.668 ± 0.19	3.22 ± 0.17	11.05 ± 0.67	10.16 ± 0.62
α-Pinene	7785-70-8	4.671 ± 0.25	4.38 ± 0.23	6.331 ± 0.35	5.55 ± 0.31	3.434 ± 0.19	3.16 ± 0.18
(+)-Longifolene	475-20-7	10.74 ± 0.63	10.08 ± 0.59	6.099 ± 0.32	5.35 ± 0.29	3.264 ± 0.17	3.00 ± 0.16
Isolongifolene	1135-66-6	9.393 ± 0.52	8.81 ± 0.48	4.356 ± 0.23	3.82 ± 0.21	6.649 ± 0.38	6.11 ± 0.35
α-Ionone	127-41-3	9.667 ± 0.56	9.07 ± 0.53	7.011 ± 0.39	6.15 ± 0.36	7.174 ± 0.41	6.59 ± 0.38
(α)-Caryophyllene	87-44-5	5.959	5.59	4.575	4.01 ±	2.110	1.94 ±

		± 0.33	± 0.30	± 0.24	0.22	± 0.11	0.09
β-Farnesene	18794-84-8	7.746 ± 0.45	7.26 ± 0.42	8.929 ± 0.50	7.83 ± 0.45	2.888 ± 0.15	2.65 ± 0.14
a-Phellandrene	99-83-2	9.134 ± 0.51	8.57 ± 0.49	9.222 ± 0.52	8.09 ± 0.47	8.015 ± 0.44	7.36 ± 0.41
(-)-Verbenone	1196-01-6	59.84 ± 3.48	56.10 ± 3.25	8.416 ± 0.48	7.38 ± 0.44	11.23 ± 0.71	10.31 ± 0.68

Table S10. Molecular docking binding site analysis

Compound name	TpiiOBP5		TpiiOBP16		TpiiOBP29	
	Hydrophobie	Van der weal	Hydrophobie	Van der weal	Hydrophobie	Van der weal
α-Pinene	Arg118、<br>Phe86、<br>Cys117、<br>Phe121、<br>Leu82、	-	Val79、<br>Phe63、<br>Ala45、<br>Ala46、<br>Phe42、<br>Val83、<br>Leu51、<br>Phe104、<br>Leu87、	Gln10、	Val84、<br>Ala47、<br>Ala101、<br>Ile98、<br>Val46、<br>Phe43、<br>Val102、<br>Phe105	Leu88、<br>His10
Camphene	Ala21、<br>Val24、<br>Ile60、<br>Phe25、	Ile22、<br>Ser42、<br>Val43、<br>His56、<br>Val24、	Phe104、<br>Val83、<br>Ala45、<br>Ala49、<br>Leu87、<br>Ala46、<br>Leu51、<br>Phe42、	Phe63、<br>Gln10、<br>Phe9、	Val102、<br>Phe105、<br>Val52、<br>Leu88、<br>Phe43、<br>Val84、<br>Val46、<br>Ala47、	Ala101、<br>Phe64、
3-Carene	Ile22、<br>Phe25、<br>Ala21、<br>Val23、	-	Phe104、<br>Phe42、<br>Ile27、<br>Phe38、<br>Tyr105、	Glu108、<br>Gly30、<br>Met28、	Val84、<br>Phe43、<br>His10、<br>Ala101、<br>Leu88、<br>Phe105、<br>Val46、	Cys87、<br>Ala47、<br>Val102、
D-Limonene	Phe66、	Lys77、	Met28、	Glu108、	Ala47、	-

	Leu62、<br>Lys81、<br>Leu78、<br>Ala114	Ser64、<br>Gly61	Tyr105、<br>Leu111、<br>Ile101、<br>Phe38、	Phe104、<br>Ile27、<br>Phe42、	Leu88、<br>Ala101、<br>Ile98、<br>Val46、<br>Val52、<br>Val102、<br>Phe43、<br>Phe105、<br>His10、<br>Phe105、	
β-Myrcene	Cys117、<br>Ala114、<br>Leu82、<br>Leu78、<br>Phe66、<br>Lys81、	Ser64、	Tyr105、<br>Phe104、<br>Phe42、<br>Phe38、	Met28、<br>Ile27、<br>Gly30、<br>Glu108、<br>Ile101、	Phe43、<br>His10、<br>Val84、<br>Val52、<br>Val46、<br>Ala47、<br>Leu88、<br>Phe43、<br>Phe105、	Val102、<br>Ala101、
Longifolene	Leu82、<br>Phe121、<br>Cys117、<br>Phe86、<br>Arg118、	Ile85、<br>Glu11、	Phe104、<br>Ile101、<br>Tyr105、<br>Phe42、<br>Met28、<br>Phe38、<br>Ile27、<br>Leu24、	His41、<br>Gln10、	Ala47、<br>Leu88、<br>His10、<br>Ala50、<br>Val52、<br>Phe64、<br>Val80、<br>Val84、<br>Val46、	Ala101、<br>Val102、<br>Ile72、
E-Caryophyllen	Phe86、	Glu11、	-	-	Phe64	Ile72

e	Cys117、Leu82、Phe121、Arg118、	Ile85、	Met68 Phe9 Ala50 Val84、Val52、Val46、Leu88、Phe43、Ala47、Phe105、Val102、Ala101、	Val80
